# Climate Warming Drives the Breakdown of Plant–Pollinator Mutualisms Despite Phenological Synchrony

**DOI:** 10.1111/gcb.71074

**Published:** 2026-08-26

**Authors:** Natasha de Manincor, Alessandro Fisogni, Oscar Godoy, Nicole E. Rafferty

**Affiliations:** ^1^ Department of Evolution, Ecology and Organismal Biology University of California, Riverside Riverside California USA; ^2^ CEFE, CNRS, University of Montpellier, EPHE, IRD Montpellier France; ^3^ Estación Biológica de Doñana, Consejo Superior de Investigaciones Científicas (EBD‐CSIC) Sevilla Spain; ^4^ School of BioSciences The University of Melbourne Parkville Victoria Australia

**Keywords:** climate change, ecological communities, fitness, indirect effects, phenology, piecewise structural equation modeling, plant–pollinator interactions, temporal synchrony, traits

## Abstract

Climate warming threatens biodiversity, driving population declines and endangering species with extinction. The disruption of species interactions and associated loss of ecological function can precede extinction, yet the effects of warming on interactions remain unresolved. Stress‐induced changes in traits and phenological mismatch between interacting species are hypothesized to be primary mechanisms by which warming threatens biodiversity. However, we lack experimental evidence of warming‐induced interaction disruption, making it difficult to gauge the importance of these trait‐based mechanisms of interaction loss. Using experimental plant–pollinator communities, we show that springtime warming weakens the mutualism not by reducing phenological synchrony but by altering multiple physiological and behavioral traits, ultimately depressing both plant and bee fitness. Despite warming‐induced phenological shifts in flowering and bee activity, temporal synchrony between partners was maintained. However, warming reduced flower abundance and bee survival, floral rewards, and bee visits to flowers. Our results refocus attention away from phenological disruption, towards physiological and behavioral changes that reduce mutualism strength in a changing climate.

## Graphical abstract

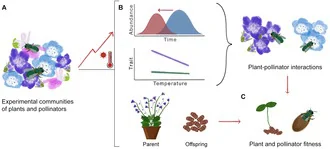

## 1 Introduction

Climate warming is driving significant changes across ecosystems worldwide, resulting in local extinctions and novel communities (Jaureguiberry et al. 2022; Pandolfi et al. 2020). The loss of species interactions precedes species loss and often occurs at a faster rate (Valiente‐Banuet et al. 2015). As temperatures rise, increasing physiological stress (Hammond et al. 2020) and alterations in phenology (Menzel et al. 2006), behavior (Hammond et al. 2020; Tuomainen and Candolin 2011), and traits (Martén‐Rodríguez et al. 2025; Soudzilovskaia et al. 2013) can disrupt species interactions and potentially diminish ecosystem stability (Fussmann et al. 2014; Thébault and Loreau 2005). Disruption of the interactions that are critical to ecosystem functioning and global food security, including the mutualistic interactions between plants and pollinators, poses a major threat (Díaz et al. 2019; Memmott et al. 2007). Understanding the extent and mechanisms of warming‐driven impacts on plant–pollinator mutualisms is crucial for assessing future risks from climatic change and developing effective conservation strategies (Forrest 2015).

Much of our knowledge of how climate change will affect ecological communities derives from studies focused on species, functional groups, and their assembled networks (Descombes et al. 2020; Martén‐Rodríguez et al. 2025; Pérez‐Ramos et al. 2019; Schleuning et al. 2016), without exploring the interspecific links that bridge these units of analysis (Gilman et al. 2010; Schleuning et al. 2020). Although these approaches can reveal the direct effects of warming on focal species (Bernhardt et al. 2018) and overall network properties (Yuan et al. 2021), they may fail to capture effects emerging from dynamic traits. These traits play a crucial role in shaping the exchange of resources and services between interacting species (Abrams 1995; de Andreazzi et al. 2020). As traits change, so may interaction costs and benefits (Magnoli et al. 2023). Although species‐level warming‐induced trait shifts are well‐documented, their effects on interactions remain unclear. Because interactions can modify the effects of climate change on species (Gilman et al. 2010), and their loss can lead to the collapse of ecosystem functions (Valiente‐Banuet et al. 2015), it is essential to know how climatic changes affect interactions themselves to predict the impacts of global warming. Yet, few studies explore the emergent effects of climate change on mutualistic interactions, whose net benefits may hinge on warming‐sensitive traits (Cruz et al. 2023), focusing instead on responses of one of the two partners (i.e., either plants or pollinators; Gérard et al. 2022; but see Hemberger et al. 2023; Traine et al. 2024).

**FIGURE 1:**
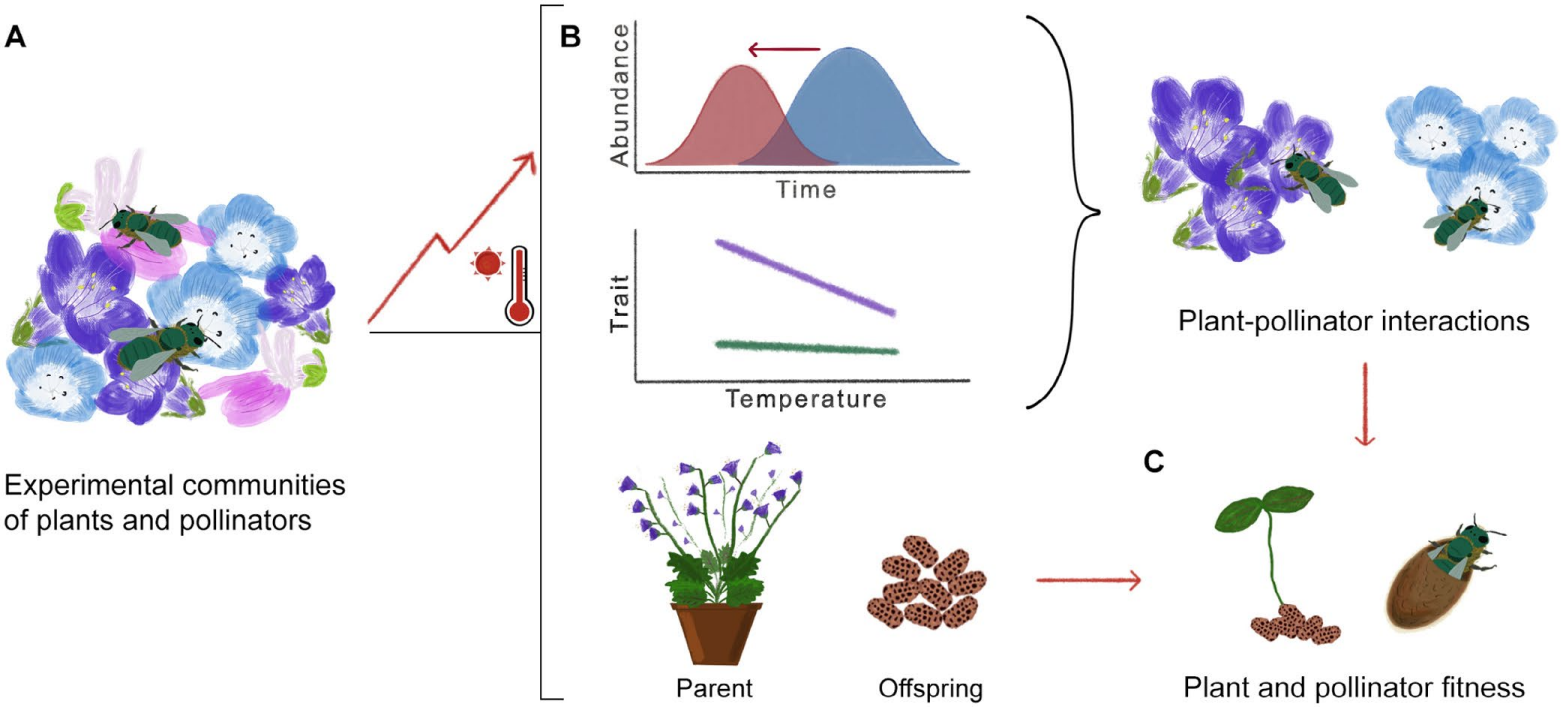
Conceptual model of expected effects of warming on experimental communities of plants and pollinators. Direct effects of warming on abundance, phenology, physiology, and behavior influence the quantity and quality of plant–pollinator interactions, while both interaction strength and parental effects influence plant and pollinator fitness components. (A) The experimental communities comprise three annual wildflower species ( *Phacelia campanularia* in purple, *Nemophila menziesii* in blue, and *Collinsia heterophylla* in pink) and one solitary bee species ( *Osmia lignaria* ) that are subjected to warming trajectories that begin prior to flowering and emergence. (B) The dynamic responses of each species are measured, capturing temporal shifts in phenological abundance curves under ambient (blue) and warmed (red) conditions, warming‐driven changes in physiological and behavioral traits, such as nectar volume (purple line representing a plant trait) and flower handling time (green line representing a bee trait). These responses influence bee visits to flowers and the quality of pollination services and floral rewards exchanged in this service‐resource mutualism. Together with warming‐induced parental effects that alter offspring quality (represented by maternal plant and seeds), interaction costs and benefits affect (C) plant and pollinator reproductive success (represented by seedling and cocoon). Illustration credit: AF.

Here, we investigate the impacts of different warming trajectories on plant–pollinator mutualisms, and the cascading effects of altered species traits and biotic interactions on plant and pollinator fitness. Using experimental communities of annual wildflowers and solitary bees, we simulated late‐winter and sustained springtime warming by imposing elevated temperatures before flowering and bee emergence and continuing through fruiting and nesting, to evaluate seasonal effects on ecological interactions. Adopting a two‐fold approach, we first captured how plant–pollinator communities dynamically responded to increasing temperatures, quantifying changes in the phenological, morphological, and behavioral traits that structure the interactions between plants and pollinators over time (Figure 1). Then, we assessed how these warming‐induced responses affected plant and bee fitness, considering both direct parental effects and indirect consequences for progeny mediated through species interactions and traits (Figure 1). This comprehensive experiment represents a significant advance over previous work that examined the direct effects of qualitative warming on individual traits and interaction rates but did not capture the dynamics or complex indirect effects within plant–pollinator communities (de Manincor et al. 2023; Magnoli et al. 2023; Martén‐Rodríguez et al. 2025). In this study, we built upon past findings with a greenhouse experiment that enabled us to dissect the trait‐based mechanisms underlying emergent effects on the mutualism. This integrated approach allows for a deeper understanding of how warming will reshape species interactions and fitness in mutualistic communities.

## 2 Materials and Methods

### 2.1 Experimental Warming

We created four temperature treatments in separate 25 m^2^ greenhouse rooms at the Plant Research 1 (PR1) facility at the University of California, Riverside, USA. All rooms had the same orientation and sun exposure. We programmed one room to simulate historic mean day/night temperatures recorded in Riverside, CA, based on monthly climate normals from 1991 to 2020 (NOAA), and three rooms to simulate different global warming scenarios. Thus, temperature setpoints in all rooms increased monthly according to the climate normals, and the warming treatment setpoints were relative to the ambient treatment setpoints (Table S1). Temperatures automatically switched from day to night based on monthly average times of sunrise and sunset, and were set and monitored at the room level to provide a baseline. We obtained an average temperature increase of +1.4°C, +2.5°C, and +6°C throughout the duration of the experiment in the three warmed rooms, respectively (Table S1). An increase of +1.4°C falls within the maximum recommended threshold to avoid large climate change risks for natural and human systems (IPCC 2018). Temperature increases of +2.5°C and +6°C are expected to cause moderate to extreme climatic events, potentially increasing the risk of irreversible changes on biodiversity and ecosystems, including species loss and extinction (Fan et al. 2020; IPCC 2018 ). To track the dynamic changes in monthly mean temperatures throughout the experiment, we placed one datalogger (Onset HOBO, Bourne, MA, USA) in each of the six arenas per room (see Section 1.2) that automatically recorded temperature every 10 min, to account for fine‐scale variability and to allow us to use actual temperatures measured in each replicate arena throughout the duration of the experiment as continuous, arena‐specific explanatory variables in the models. Our warming treatments were imposed once plants and bees were synchronized in their development and placed together into experimental foraging arenas. Specifically, we initiated warming 10–14 days prior to flowering onset (based on floral bud formation) and bee emergence (based on data on emergence latency relative to wintering duration and incubation temperature; Bosch and Kemp 2001). With this approach, if plants and bees respond similarly to warming, their phenologies should remain synchronous; any phenological divergence can be attributed to warming.

### 2.2 Plant Communities

Within each of the four rooms we set up six fine‐mesh arenas (Even Naturals Mosquito Tents; 1.6 m long × 1.6 m wide × 1.8 m high). Five arenas were used to create experimental plant–pollinator communities (hereafter: foraging arenas), while the sixth contained only plants that were used to measure flower traits to avoid disturbing pollinator activity (hereafter: trait arenas). Plant communities consisted of three annual spring forbs native to California: *Collinsia heterophylla* (Plantaginaceae), *Nemophila menziesii* (Boraginaceae), and *Phacelia campanularia* (Boraginaceae). All three species are self‐compatible, although floral morphology and development reduce autonomous autogamy and promote outcrossing (Armbruster et al. 2002; Cruden 1972; Gillett 1961). All species produce nectar and pollen, are highly reliant on pollinators for seed production, and are visited by *Osmia lignaria* (Boyle et al. 2020; Wood 2010). Plants were grown from seeds purchased from a local plant nursery (Theodore Payne Foundation). In December 2021, we planted 432 seeds per species in peat pellets (Jiffy, Lorain, OH, USA) in individual pots to promote germination and early growth under ambient greenhouse conditions (mean 13°C–16°C, maximum 20°C–24°C, minimum 6°C–8°C; natural photoperiod, watered ad libitum). At the end of January/beginning of February 2022, we transferred 400 seedlings per species to 1.9 L pots (with drainage holes) filled with a standard soil mixture (57% sand, 43% peat moss, and various minerals), applied 5 g of slow‐release fertilizer (Osmocote, Charleston, SC, USA), and watered them ad libitum. On February 28th, we individually labeled and numbered 1080 plants presenting at least 1–2 unopened flower buds but without any open flowers, indicating they were at the same developmental stage prior to our temperature treatments. On the same day, we haphazardly transferred 90 plants per species into each room, 15 per arena (i.e., 45 plants in total per arena, 270 plants per room). Plants were haphazardly arranged in nine trays of five plants each, with 1–2 individuals per species per tray, and were automatically watered via individual drip stake emitters once a day. Plants were watered equally across all treatments and arenas using automatic drip irrigation programmed to deliver the same volume of water per pot per unit time (approximately 50 mL per day), at rates such that plants in the warmest communities were not wilted or otherwise visibly droughted, and allowing for species‐specific covariation between temperature and soil moisture.

### 2.3 Pollinator Communities

The pollinator in our experiment was the blue orchard bee, *Osmia lignaria* (Hymenoptera, Megachilidae), a species native to North America, including southern California. We chose this species as a model because it shares many life history traits (e.g., solitary, univoltine, polylectic, and cavity‐nesting) with a high proportion of wild bees (Dorian et al. 2023; Slominski and Burkle 2019), and it is an important pollinator for both wild and agricultural plant species (Bosch and Kemp 2001; Boyle et al. 2020). In the spring, females lay eggs that they provision with pollen and nectar in pre‐existing cavities; eggs usually hatch within a few days, and larval development is typically completed in the summer; larvae develop from pre‐pupae into pupae by late summer, and by the fall pupae develop into adults which undergo a winter diapause before emerging the following spring (Bosch and Kemp 2001; Sgolastra et al. 2010). Development and emergence phenology are influenced by temperature (Sgolastra et al. 2011; Slominski and Burkle 2019). We obtained 420 cocoons (168 females: 252 males) on February 8th, collected in late summer 2021 from natural habitats at the foothills of the Sierra Nevada mountains, CA (Foothill Bee Ranch, USA). Cocoons were overwintered at the ranch for about 90 days at 4°C and 70% relative humidity (RH), and for another 17 days at 4°C and 70% RH in a cold room at PR1. Cocoons were weighed in the cold room on February 25th to randomize weights among the arenas and temperature treatments. On March 2nd, we placed 10 cocoons into each of the five foraging arenas in each of the four rooms, with a female: male ratio of 2:3 (i.e., four females and six males) to match the recommended sex ratio for *O. lignaria* (Bosch and Kemp 2001) and obtain full plant–pollinator communities. Cocoons were inserted in 15 cm long × 7.5 mm diameter paper straw liners plugged on one end, placed on top of artificial nests, and bees were allowed to emerge autonomously in the four temperature treatments. Cocoons were placed into foraging arenas in the evening to avoid temperature shocks. Artificial nests consisted of seven 15 × 10.5 cm overlapping wooden boards containing a total of 48 empty paper straw liners (same size as the ones containing cocoons, plugged on one end) arranged in six rows for bees to rest between flights and for nesting, placed in a wooden shelter (Knox Cellars Mason Bees) at a height of 50 cm on one side of the arena facing south. We supplied a constantly moist clay‐soil mixture in small trays placed at the base of the artificial nests for nest cell construction (Bosch and Kemp 2001).

### 2.4 Plant Phenology

We checked plants daily starting on March 3rd, and recorded the date of first bloom for all plants in all arenas. We then counted the number of open flowers for the same two plants per species per arena (i.e., six plants per species per arena, 36 plants per room, 144 plants in total in the four rooms) three times a week on non‐consecutive days, from flowering onset until all plants died (June 1st).

### 2.5 Flower Traits

We measured flower size and nectar production in trait arenas twice a week on non‐consecutive days, alternating morning and afternoon sampling to account for daily variability in resource production. On each sampling date, we haphazardly selected two open flowers per species on different plants, for a total of four flowers per species per week. We sampled each flower only once, and avoided sampling the same plant in consecutive weeks, for a total of 243 flowers over the four temperature treatments and experiment duration (62 flowers in the ambient room, 60 in the +1.4°C room, 61 in the +2.5°C room, and 60 in the +6°C room). For each flower, we first measured corolla size as a proxy for visual floral attractiveness using digital calipers (Neiko, Taiwan). We measured two perpendicular diameters in actinomorphic flowers (i.e., *N. menziesii* and *P. campanularia* ), and keel width and banner height in the zygomorphic flowers of *C. heterophylla* (see Figure 1 and de Manincor et al. (2023) for graphic representations). We approximated actinomorphic corolla surface to an ellipse area, and zygomorphic corolla surface to twice an ellipse area to account for both the keel and banner surface (modified from Knauer and Schiestl (2015)). Then, we extracted all of the available nectar with glass microcapillary tubes (Drummond Scientific Co., Broomall, PA, USA) and measured the nectar column length using digital calipers to quantify the nectar volume. Because nectar volume and concentration were highly negatively correlated (Pearson's correlation coefficient = −0.83, *p* value < 2.2e^−16^), here we used only volume as a proxy for resource availability (see de Manincor et al. (2023) for methodology on nectar concentration). Flower trait measurements started on March 15th and ended on April 21st. On the same dates and on the same plants used for measuring flower traits, we recorded soil volumetric water content (% VWC) using a handheld soil moisture sensor (Campbell Scientific, HydroSense II, Logan, UT, USA), to estimate the ratio of the volume of water to the unit volume of soil.

### 2.6 Pollinator Phenology

We visually checked artificial nests in all foraging arenas twice a day from March 3rd to assess successful bee emergence. Each sampling day we recorded the number of active bees, and at the end of the day we checked arenas for dead bees until all bees were dead. The last bee died on May 9th. Because all 10 cocoons allocated to each arena were drawn from the same source population and treated identically prior to placement in the arenas, we can attribute differential survival (whether due to differential emergence or post‐emergence lifespan) to the temperature treatments. We report bee abundance to indicate how many bees were alive at a given time point; differences in abundance translate to differences in survival (and mortality).

### 2.7 Plant–Pollinator Interactions

On the same days of flower counting, we observed plant–pollinator interactions. Two of us (NdM and AF) conducted simultaneous 10‐min observations of foraging arenas in the morning (starting at 10 AM) and in the afternoon (starting at 2 PM) in different rooms, for a total of 100 min per room per day (i.e., 400 min in total per day over the four rooms). Observations consisted of visually following one bee at a time until the end of its foraging bout (i.e., male or female bee flights seeking nectar and/or pollen, which ended when bees rested in nests or on arena walls), recording the number of flowers visited and the duration of visits per flower. The order of observations (arena, room, AM/PM) was haphazardly switched at every observation round. Interactions were recorded for 27 days over a 9‐week period, for a total of 10,800 min of observations.

### 2.8 Plant Reproductive Output

At the end of the flowering period, we collected 10 mature undehisced fruits from each of the six plants used for flower counting in foraging arenas, 2–7 days apart (i.e., 100 fruits per species per room, 300 fruits per room, 1200 fruits in total for the four rooms). Fruits were stored in paper bags until dried at 60°C for 24 h in a drying oven (Shel Lab SG03). Two haphazardly chosen fruits per plant (i.e., 20 fruits per species per room, 60 fruits per room, 240 fruits in total for the four rooms) were then each opened under a dissecting microscope (Zeiss Stemi 508), and the number of seeds per fruit and undeveloped ovules were counted. All seeds from the same fruit were then weighed to the nearest 0.0001 g (Mettler Toledo MS104TS). Total seed mass was divided by the number of seeds in each fruit to obtain average seed mass.

### 2.9 Seed Germination

To check for plant parental effects on the F1 generation, we haphazardly chose two seeds from additional mature fruits that had been harvested about 12 months prior and stored in paper bags at about 20°C until germinated. Each set of two seeds came from three different plants per species per arena (i.e., 30 seeds per species per room, 90 seeds per room, 360 seeds in total for the four rooms). Each seed was planted in an individual peat pellet placed within 36‐well inserts in trays. Trays were placed in a common growth chamber (Percival PGC‐40L2) in PR1 at 20.8°C—30% RH during the day and 6.7°C—40% RH during the night, with daily settings simulating mean day/night temperatures and light/dark cycles (sunrise: 6:50 AM, sunset: 4:50 PM) for the month of January in Riverside. Seeds were watered and checked daily until germination or until the end of the experiment. Seeds were considered successfully germinated when a radicle was visible. The dates on which seedlings developed cotyledons and true leaves were noted, as was seedling death at any point. Observations lasted 30 days, ending when 10 days had elapsed since any seeds germinated.

### 2.10 Bee Reproductive Output

After all bees died, we left nests in the foraging arenas for 4 weeks to allow egg and larval development under the same temperatures experienced by adults. We then checked all paper straw liners for completed nests and counted the number of cells in each nest (delimited by mud partitions) and the number of fully developed cocoons in each cell. We recorded a total of 137 nest cells and 28 cocoons throughout the 20 arenas.

### 2.11 Data Analysis

All data produced in this experiment (de Manincor et al. 2025) were analyzed in the R statistical environment (v.4.5.1) (R Core Team 2025). All model residuals were checked using the R‐package *DHARMa* (Hartig et al. 2024).

#### 2.11.1 Experimental Warming

To assess average temperature differences obtained in the four experimental rooms over time, we performed repeated measurement ANOVAs on mean daily temperatures recorded in each of the six arenas within the four rooms throughout the duration of the experiment. After comparing temperatures in the three rooms simulating different warming scenarios to the room set at ambient temperatures (main model intercept), we performed all pairwise comparisons to check for consistent and significant differences in temperatures between all rooms throughout the experiment. Model coefficients are reported in Table S1. Models were fit using R‐packages *lme4* (Bates et al. 2015) and *emmeans* (Lenth 2024). To check for covariation between temperature and soil moisture, we fitted a beta regression (R‐package *glmmTMB*, Brooks et al. 2017) using % VWC as the response variable and temperature at the arena level as the continuous explanatory variable. Day of year (to account for temporal autocorrelation) and plant species (to account for species‐specific responses at the plant community level) were included as random factors. Furthermore, we fitted separate beta regressions for each plant species using % VWC as the response variable, continuous temperature as the explanatory variable and day of year as a random effect to check for species‐specific covariance levels.

#### 2.11.2 Phenological Distributions

To characterize changes in plant and pollinator phenological distributions in relation to temperature, we quantified the mean, skewness, and breadth for arena‐level phenological abundance distributions (Stemkovski et al. 2023), using the number of open flowers and surviving bees at each sampling day in each foraging arena. We calculated skewness as the Fisher‐Pearson standardized third‐moment coefficient of skewness, and breadth as the estimator of Pearson's measure of kurtosis, using the R‐package *moments* (Komsta and Novomestky 2022). Then, we fitted separate linear models using distribution mean, skewness, and breadth as response variables, and organism type (flowers vs. bees), average temperature calculated for each arena over the duration of the experiment, and their interaction as continuous explanatory variables.

#### 2.11.3 Phenological Shifts

To assess if plant and pollinator phenology shifted in relation to warming temperatures, we fitted two generalized linear mixed‐effect models (GLMMs; R‐package *glmmTMB*, Brooks et al. 2017). We used daily flower and bee abundance at the arena level as response variables, respectively, and temperature (continuous variable), day of year, day of year squared (to account for non‐linear effects), and their interaction as explanatory variables. Arena identity (to account for the experimental block design) and day of year (to account for temporal autocorrelation) were included as random effects. We used negative binomial (log‐link) and Poisson (log‐link) error distributions for plant and pollinator phenology models, respectively.

#### 2.11.4 Phenological Overlap

To assess if the overlap of activity patterns changed in relation to temperature, we estimated phenological overlap of plant and pollinator abundance distributions at the arena level (Ridout and Linkie 2009). First, we converted day of year to radians and fitted non‐parametric von Mises kernel density functions to daily flower and bee abundances in each arena over the duration of the experiment. Then, we calculated overlapping coefficients, ranging between 0 (no overlap) and 1 (complete overlap), by estimating the area shared by the two functions (R‐package *overlap*, Meredith et al. 2024). Finally, we fitted a beta regression (R‐package *glmmTMB*, Brooks et al. 2017) using the estimated plant and pollinator overlapping coefficients as response variables, and average temperatures squared (to account for non‐linear effects) at the arena level as continuous explanatory variables.

#### 2.11.5 Piecewise Structural Equation Models

To evaluate direct and indirect effects of increasing temperatures on plant and pollinator traits, their interactions, and plant fitness, we used piecewise structural equation models (pSEMs). Piecewise SEMs allow for the fitting of linear models with different error distributions (i.e., local estimations), summarizing causal relationships between the measured experimental variables using a priori knowledge of the system (Lefcheck 2016; Shipley 2009). We evaluated the independence between variables through tests of directed separation, and assessed pSEMs goodness‐of‐fit by using Fisher's *C* statistics and the associated *p* values, with *p* values > 0.05 indicating good fit of the model to the data (Shipley 2009). Separate GLMMs within pSEMs were fitted using the R‐package *glmmTMB* (Brooks et al. 2017). Piecewise SEMs were fitted using the R‐package *piecewiseSEM* (Lefcheck 2016).

#### 2.11.6 Dynamic pSEM

First, we fitted a pSEM (hereafter: dynamic pSEM) on variables recorded throughout the duration of the experiment (i.e., number of flowers, number of bees, number of flower visits, flower handling time, flower size, and nectar volume), to evaluate dynamic changes in continuous relationships between plants and pollinators influenced by temperature over time. Because some variables were recorded on different days and plants, we performed data imputations before fitting the dynamic pSEM to fill in missing data (Blomberg and Todorov 2025), and allow for a matched comparison between all variables included in the model. We used the R‐package *Amelia II* (Honaker et al. 2011) to obtain 100 imputations of our incomplete multivariate dataset, based on expectation–maximization (EM) algorithms. We incorporated time series in the imputation models as second‐order polynomials to allow for non‐linear effects over time, and included cross‐sectional units to allow for all imputed variables to vary across temperatures and plant species (Honaker and King 2010). We also included a ridge prior of 1% to increase numerical stability by shrinking the covariances of the data without changing the means or variances (Honaker et al. 2011). All variables were log‐transformed before imputations to fit the multivariate normal distribution assumptions of the model (King et al. 2001). Imputations were checked for normal EM chain convergence, and goodness‐of‐fit was evaluated by comparing imputed data distributions to original data distributions. We then averaged each variable over the 100 imputed datasets before using them in the dynamic pSEM. The average number of flowers and bees were rounded to the nearest integer. Model paths were included based on biological and ecological expectations, as detailed in Table S2. Overall, we evaluated direct effects of increased temperature on bee and flower abundance, as well as on flower traits that could affect attractiveness to pollinators, and the cascading effects they had on the patterns of flower visits (Figure 1). Individual model paths were fitted using GLMMs with either Poisson (log‐link) or Gaussian (identity‐link) error distributions, and included day of year (to account for temporal autocorrelation), plant species (to account for species‐specific responses at the plant community level) and arena identity (to account for the experimental block design) as random effects, depending on the response variables (Table S3). We specified non‐causal error correlations in the pSEM model to fit conditional independence claims (Table S4). The model fit the data well based on Fisher's *C* statistic (*C*_6_ = 2.49, *p* = 0.29).

#### 2.11.7 pSEM for Plant Fitness

Because seeds were collected only once at the end of the experiment, we performed a pSEM to include direct and indirect effects of temperature on plant fitness. Using the imputed datasets, we calculated average values at the arena level for all variables included in the dynamic pSEM that could potentially affect plant fitness over the entire duration of the experiment. To add the fitness component, we included the number of total ovules and mature seeds produced by the sampled fruits, the average seed mass, the number of germinated seeds, and the number of seedlings that died before developing cotyledons as response variables in the linear paths (Table S3). Models included plant species (to account for species‐specific responses at the plant community level) or arena identity (to account for the experimental block design) as random effects, depending on the response variable (Table S3). Because flower size and the duration of flower visits did not have significant effects on plant fitness, we excluded these variables from the final model. We also excluded seed mass from the final model because there were no significant paths related to it. We specified non‐causal error correlations in the pSEM model to fit conditional independence claims (Table S4). As before, the model fit the data well (*C*_14_ = 12.79, *p* = 0.54).

#### 2.11.8 pSEM for Bee Fitness

As with the pSEM for plant fitness, we performed a pSEM to include direct and indirect effects of temperature on bee fitness, which was measured only once at the end of the experiment. Using the imputed datasets, we calculated average values at the arena level for all variables included in the dynamic pSEM that could potentially affect bee fitness over the entire duration of the experiment. To add the fitness component, we included the number of nest cells built by adult bees and the number of cocoons retrieved in each arena (Table S3). Models included plant species (to account for species‐specific responses at the plant community level) or arena identity (to account for the experimental block design) as random effects, depending on the response variable (Table S3). We specified non‐causal error correlations in the pSEM model to fit conditional independence claims (Table S4). As before, the model fit the data well (*C*_20_ = 23.7, *p* = 0.25).

## 3 Results

### 3.1 Effects of Warming on Plant and Pollinator Phenology

**FIGURE 2:**
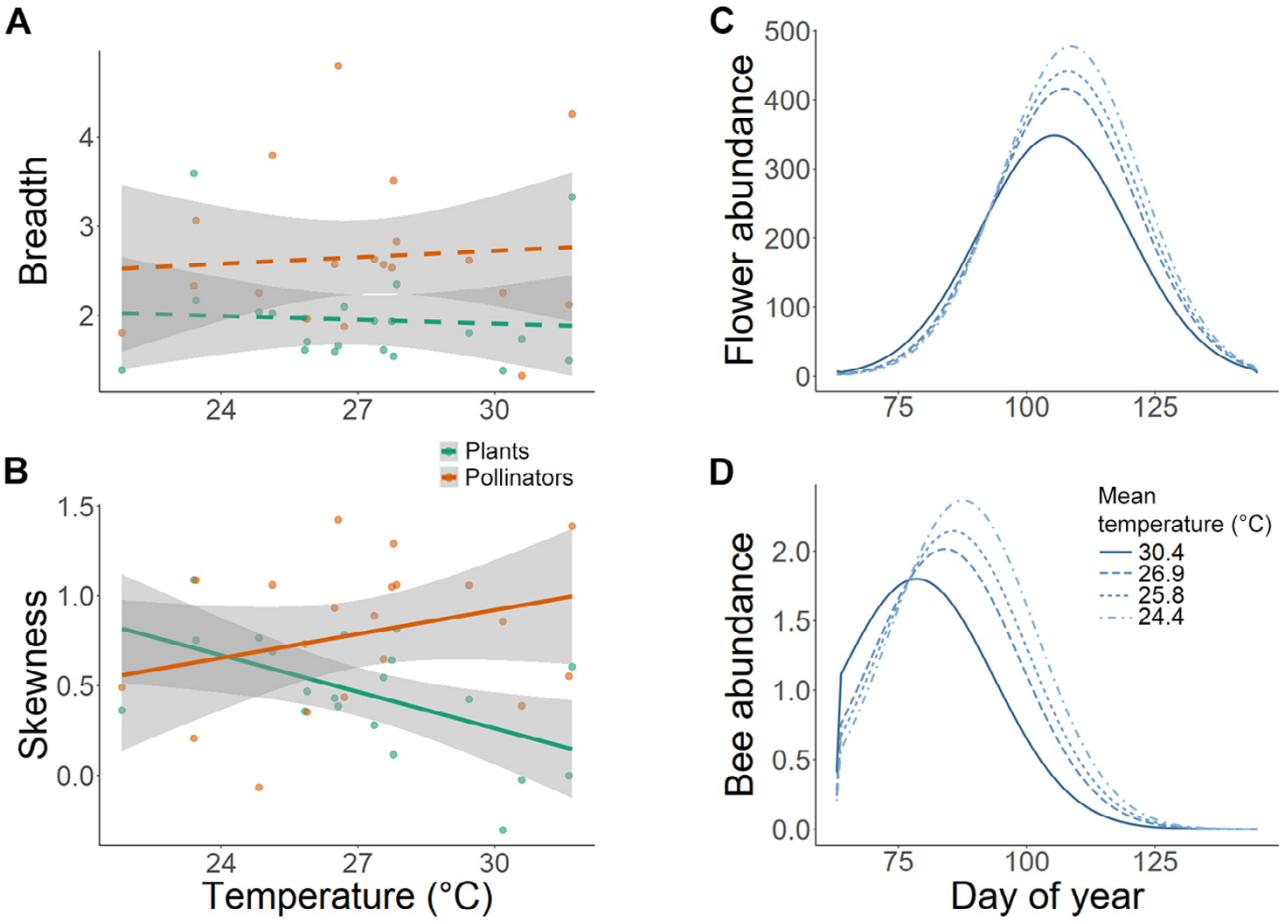
Plant and pollinator phenological distributions. Interaction plots for (A) breadth and (B) skewness of flowering and pollinator phenological distributions, and (C) flower and (D) pollinator phenology in relation to increasing temperature. Solid lines in panels A and B indicate significant interactions, dashed lines indicate non‐significant interactions (model coefficients in Table S5). Points represent values recorded in the 20 foraging arenas over the duration of the experiment, and shaded areas represent 95% CIs. Curves in panels C and D were estimated using overall mean temperatures recorded at the room level, simulating ambient temperatures (24.4°C) and three warming scenarios (+1.4°C, +2.5°C, and +6°C). CIs were omitted for clarity (full coefficients are reported in Table S6).

Warming reduced flowering and bee foraging activity throughout the study, as indicated by negative relationships between the mean abundances of flowers and bees over time and temperature (Figure S1, Table S5). The breadth of plant and bee phenological abundance curves was not affected by temperature (Figure 2A), although warming had opposing effects on their skewness, with curves becoming less right‐skewed for plants (fewer flowers produced later) and more right‐skewed for bees (more bees active later) at higher temperatures (Figure 2B, Table S5). Warming also caused greater advances in bee emergence and foraging phenology than in flowering phenology (Figure 2C,D, Table S6). The amount of temporal overlap between flowering and foraging was not affected by temperature (Table S7).

### 3.2 Effects of Warming on Plant and Pollinator Abundances and Traits

**FIGURE 3:**
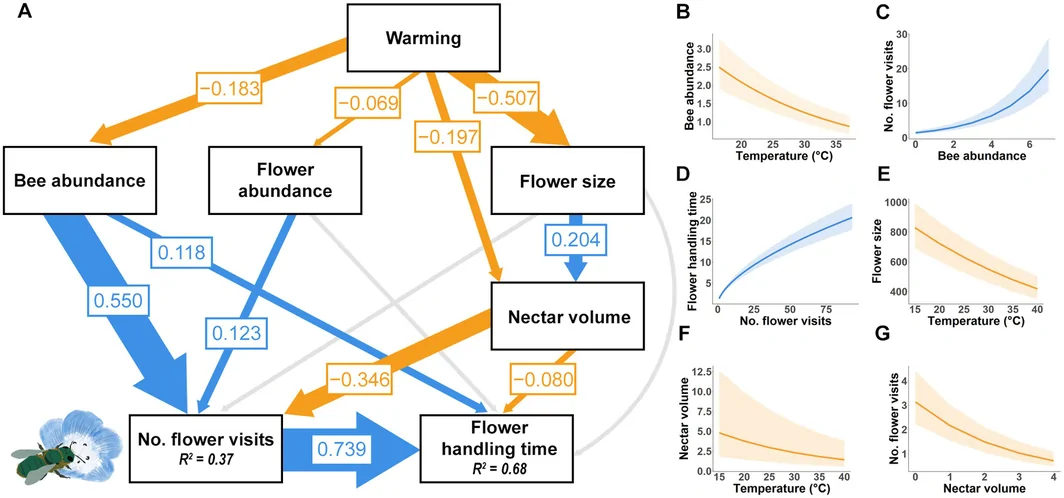
Piecewise structural equation model (pSEM) describing the dynamic effects of warming on plant and pollinator communities. (A) The direct and indirect effects of warming over time on the number of flower visits and flower handling time. Regression plots (predicted average effects and 95% CIs) highlight the direct effects of (B) temperature on bee abundance, (C) bee abundance on number of flower visits, (D) number of flower visits on flower handling time, (E) temperature on flower size (mm^2^), (F) temperature on nectar volume (μL), and (G) nectar volume on number of flower visits found in the pSEM. Arena‐level daily averages are used for all variables, except bee and flower abundances, which use daily totals. Orange arrows and regression lines represent negative effects; blue arrows and regression lines represent positive effects; gray arrows represent non‐significant effects. Standardized coefficients for each direct effect (A) are reported in boxes over the arrows. Arrow widths are proportional to the standardized path coefficients. *R*‐squared values report the proportion of variance in number of flower visits and flower handling time (i.e., time spent foraging on flowers, in seconds) explained by the pSEM. Number of flower visits and flower handling time were back‐transformed to the original values for clarity.

The dynamic pSEM showed that warming had direct negative effects on all tested variables (Figure 3A, Table S8). Warming reduced bee survival (pSEM path coefficient = −0.183, Figure 3A,B, Table S8) and flower abundance (−0.069, Figure 3A, Table S8). Lower bee (0.550, Figure 3A,C, Table S8) and flower (0.123, Figure 3A, Table S8) abundances resulted in less‐frequent flower visits (total effect = 0.673, Figure 3A), indicating that warming indirectly reduced the strength of the mutualism (indirect effect = −0.109, Figure 3A). Both higher bee abundance (0.118, Figure 3A, Table S8) and a higher number of flower visits (0.739, Figure 3A,D, Table S8) increased flower handling time.

Warming negatively affected plant traits in a consistent way (Figure 3A, Table S8). Warmed flowers were smaller (−0.507, Figure 3A,E, Table S8) and had lower nectar volume (−0.197, Figure 3A,F, Table S8). Warming also reduced per‐flower nectar available to bees both directly and indirectly via reduced flower size (total effect = −0.300, Figure 3A). These effects arise from the negative covariation between soil moisture and air temperature that trigger species‐specific responses within the experimental communities according to their water use tolerances (Figure S2, Table S9).

Reduced nectar volume caused bees to visit more flowers (−0.346, Figure 3A,G, Table S8), while directly increasing flower handling time (−0.080, Figure 3A, Table S8). Increased temperature indirectly increased handling time through reduced nectar volume (indirect effect = 0.016, Figure 3A). The number of flower visits was negatively affected by warmer temperatures via reduced bee and flower abundance and altered floral traits (indirect effect = −0.073, Figure 3A).

### 3.3 Effects of Warming on Plant Fitness

**FIGURE 4:**
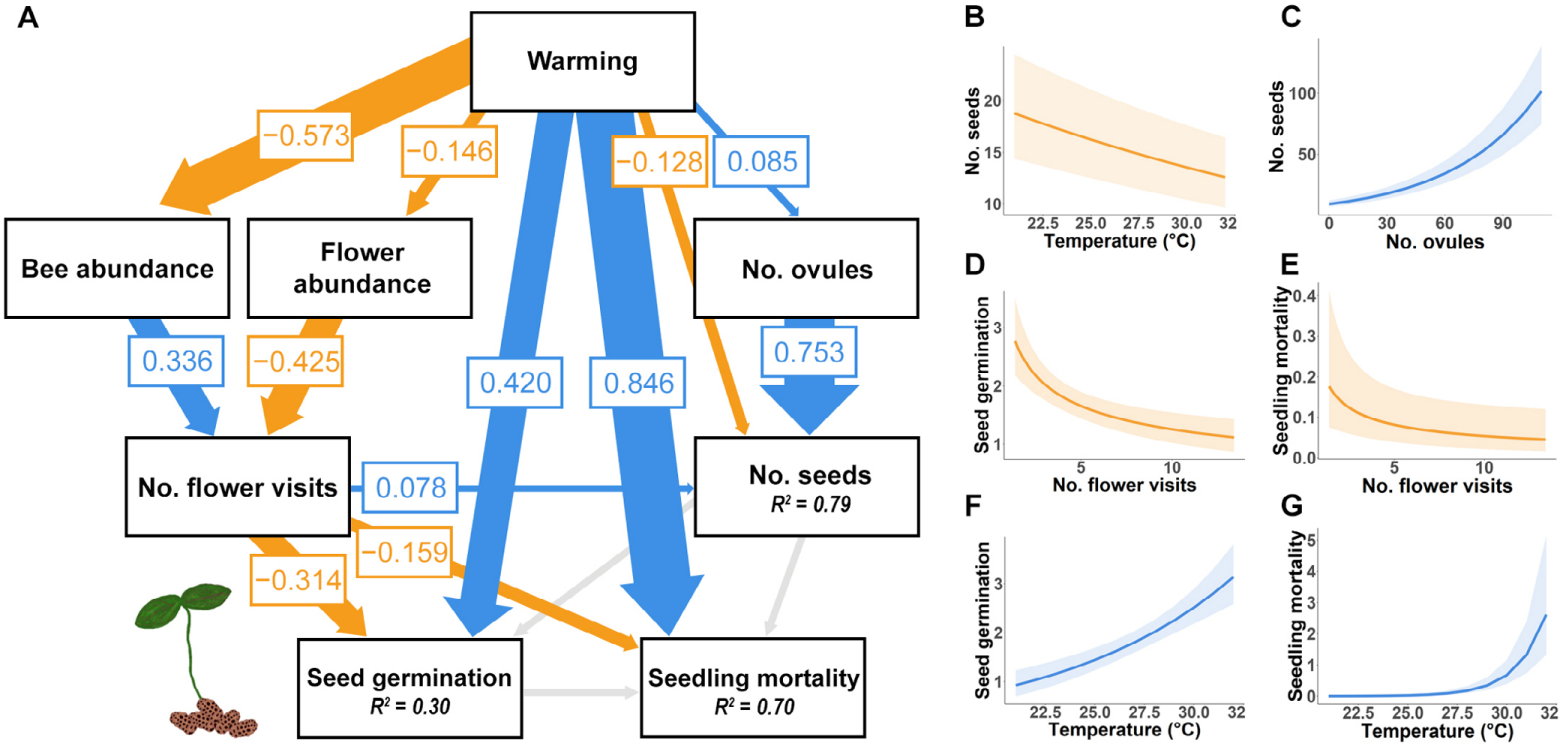
Piecewise structural equation model (pSEM) describing the effects of warming on plant fitness. (A) The direct and indirect effects of warming. Regression plots (predicted average effects and 95% CIs) highlight the direct effects of (B) temperature on number of seeds, (C) number of ovules on number of seeds, (D) number of flower visits on seed germination, (E) number of flower visits on seedling mortality, (F) temperature on seed germination, and (G) temperature on seedling mortality found in the pSEM. Arena‐level season‐long averages are used for all variables, except fitness components, which are measures from the end of the season. Orange arrows and regression lines represent negative effects; blue arrows and regression lines represent positive effects; gray arrows represent non‐significant effects. Standardized coefficients for each direct effect (A) are reported in boxes over the arrows. Arrow widths are proportional to the standardized path coefficients. *R*‐squared values report the proportion of variance in number of seeds, seed germination success, and seedling mortality explained by the pSEM. The number of flower visits was back‐transformed to the original values for clarity.

To evaluate direct and indirect effects of warming on plant reproductive fitness, we averaged bee and flower variables over the season and incorporated them in a pSEM with the number of seeds per fruit, seed germination success, and seedling mortality to capture aspects of both offspring quantity and quality (Figure 4A).

Warming directly reduced the number of seeds per fruit (−0.128, Figure 4A,B, Table S10). Thus, despite the positive effect of warming on the number of ovules per flower (0.085, Figure 4A, Table S10) and the positive relationship between number of ovules and seeds per fruit (0.753, Figure 4A,C, Table S10), warmer temperatures reduced the probability that these additional ovules developed into seeds, thereby reducing seed set (total effect = −0.065, Figure 4A).

As in the dynamic model, warming reduced the abundances of bees (−0.573, Figure 4A, Table S10) and flowers (−0.146, Figure 4A, Table S10), and indirectly depressed the frequency of flower visits (indirect effect = −0.130, Figure 4A). With more flower visits by bees in ambient communities, the number of seeds per fruit was higher (0.078, Figure 4A, Table S10). The effects of greater visit frequency on seed quality were mixed, as seeds had lower germination success (−0.314, Figure 4A,D, Table S10), while seedlings had higher survival rates (−0.159, Figure 4A,E, Table S10).

Seed germination success benefited from a direct, positive parental effect of warming (0.420, Figure 4A,F, Table S10), whereas seedlings from warmed parents suffered a larger, direct increase in mortality (0.846, Figure 4A,G). Across all paths, warming led to increased germinability of seeds (total effect = 0.846, Figure 4A), but to an even greater extent, warming resulted in increased mortality of seedlings (total effect = 1.738, Figure 4A).

### 3.4 Effects of Warming on Bee Fitness

**FIGURE 5:**
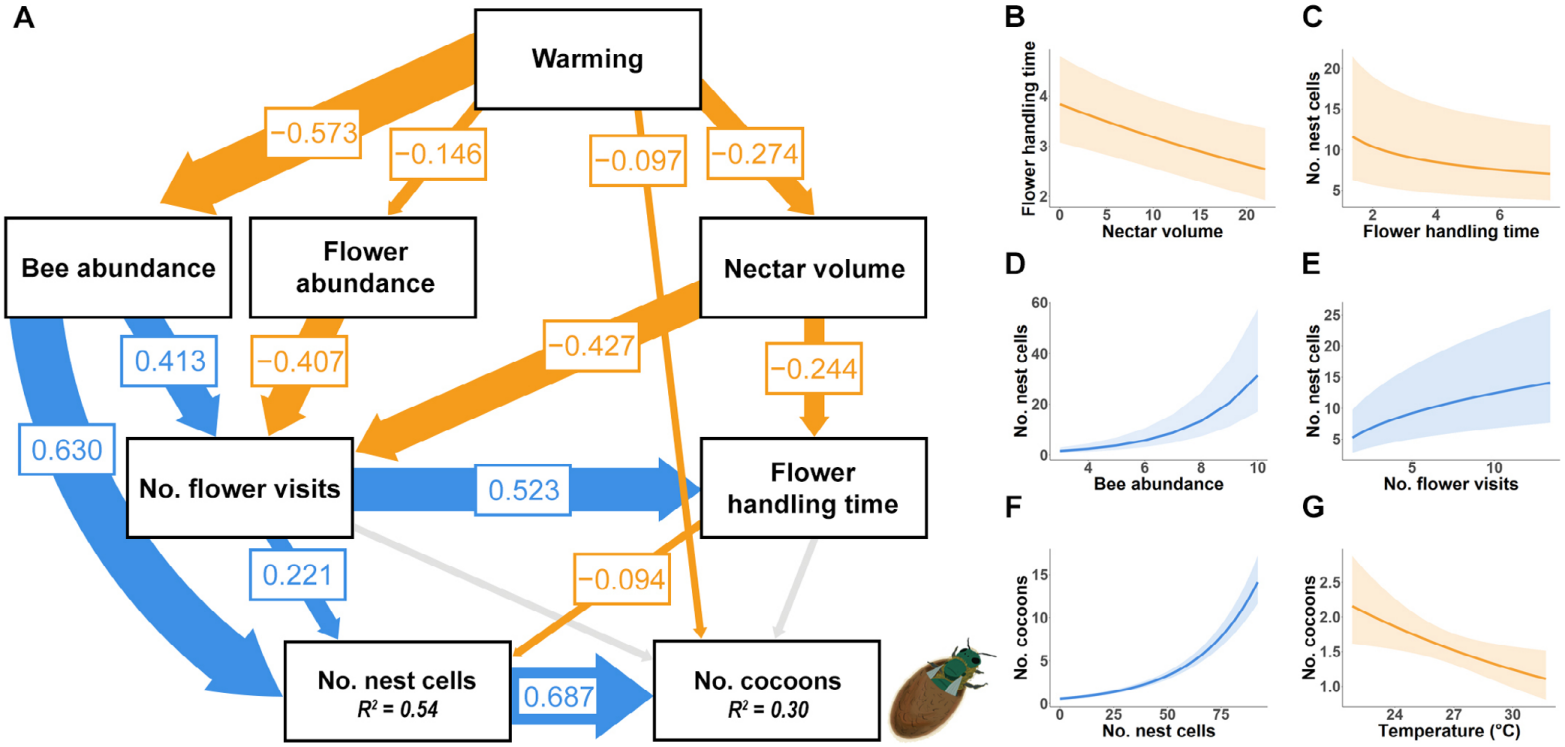
Piecewise structural equation model (pSEM) describing the effects of warming on pollinator fitness. (A) The direct and indirect effects of warming. Regression plots (predicted average effects and 95% CIs) highlight the direct effects of (B) nectar volume (μL) on flower handling time, (C) flower handling time (in seconds) on number of nest cells, (D) bee abundance on number of nest cells, (E) number of flower visits on number of nest cells, (F) number of nest cells on number of cocoons, and (G) temperature on number of cocoons found in the pSEM. Arena‐level season‐long averages are used for all variables, except fitness components, which are measures from the end of the season. Orange arrows and regression lines represent negative effects; blue arrows and regression lines represent positive effects; gray arrows represent non‐significant effects. Standardized coefficients for each direct effect (A) are reported in boxes over the arrows. Arrow widths are proportional to the standardized path coefficients. *R*‐squared values report the proportion of variance in number of nest cells and number of cocoons explained by the pSEM. Number of flower visits and flower handling time were back‐transformed to the original values for clarity.

We determined the direct and indirect effects of warming on bee reproductive fitness using an approach similar to that used for plant fitness (see details in Section 2). We averaged bee and flower variables over the season and incorporated them in a pSEM with the number of nest cells and number of cocoons to capture analogous aspects of bee offspring quantity and quality (Figure 5A).

Warming reduced bee fitness via multiple paths. First, consistent with the dynamic model, warming directly reduced nectar volume (−0.274, Figure 5A, Table S11), which in turn increased bee handling time of flowers (−0.244, Figure 5A,B, Table S11), resulting in decreased nest cell production (−0.094, Figure 5A,C, Table S11). Second, warming reduced bee survival (−0.573, Figure 5A, Table S11) and flower abundance (−0.146, Figure 5A, Table S11), which lowered the frequency of flower visits (indirect effect = −0.178, Figure 5A) and indirectly reduced nest cell production (indirect effect = −0.039, Figure 5A). Indeed, bee abundance was strongly and positively related to the number of nest cells produced (0.630, Figure 5A,D, Table S11), an effect that, when added to the direct positive effect of interaction frequency on nest cell production (0.221, Figure 5A,E, Table S11), became even stronger (total effect = 0.791, Figure 5A).

The number of nest cells was positively related to the number of cocoons (0.687, Figure 5A,F, Table S11). Separately from the effects of warming on reproductive effort via the number of nest cells, warming had a direct negative effect on the number of cocoons produced per community (−0.097, Figure 5A,G, Table S11).

Bees had longer handling times when flowers had smaller nectar volumes (−0.244, Figure 5A,B, Table S11). Thus, greater bee abundances indirectly increased handling time (indirect effect = 0.216, Figure 5A).

## 4 Discussion

Our experimental results indicate that sustained climatic warming weakens the mutualism between plants and pollinators (Figure 1). The weakening of this mutualism emerges from the dynamic physiological and behavioral responses of both partners to warming, in addition to warming‐driven reductions in flower abundance and bee survival (Figure 3), and in spite of springtime warming not altering phenological synchrony (Figure 2). All of these early effects of warming, manifested in reduced interaction rates and reduced reproductive success of both plants (Figure 4) and bees (Figure 5), may lead to mutualism loss (Kiers et al. 2010).

### 4.1 Warming Shifts Plant and Pollinator Phenology Without Altering Synchrony

Warming caused flowering to become more symmetrical around the modal date, reducing the number of flowers produced relatively late, while simultaneously causing bee emergence to be concentrated around earlier dates with a longer tail of bees active later. Despite these multifaceted changes in phenological distributions, the amount of temporal overlap between flowering and foraging was not affected by temperature. Thus, late‐winter and springtime warming, initiated when plants and bees were synchronized at the same developmental point (prior to flowering onset and bee emergence), did not induce phenological asynchrony, contrary to expectations that mutualists occupying different trophic levels are at risk of becoming mismatched due to differing reliance on and/or sensitivity to climatic cues (Thackeray et al. 2016). This maintenance of temporal overlap despite differing phenological responses of species in a common environment suggests that mismatches cannot be predicted by often‐used descriptors of phenological response, such as the rate of advance over time or phenophase sensitivity to warming (Inouye et al. 2019). Instead, it is necessary to examine how climatic changes affect multiple components of phenological abundance curves, including skewness and breadth (Stemkovski et al. 2023), and to quantify overlap between interacting species. By relating changes in the shapes of abundance‐weighted phenological distributions to climatic cues, we can better characterize the complex ways in which climate change alters phenologies and the temporal dynamics of species interactions (Fisogni et al. 2025). Yet, as we found, the maintenance of phenological synchrony under warming does not imply that mutualistic interactions remain strong.

### 4.2 Warming Weakens Plant–Pollinator Mutualisms via Dynamic Changes in Abundances and Traits

Although phenological overlap between plants and bees remained constant, warming negatively affected both partners in several ways, including by reducing bee survival (Figure 3A,B) and flower abundance (Figure 3A). These effects likely reflect warming‐induced physiological stress that culminated in greater bee mortality and depressed flower production in warmed communities (Borghi et al. 2019; Vilchez‐Russell and Rafferty 2024). Reduced bee and flower abundances resulted in less‐frequent flower visits, indicating that warming indirectly reduced the strength of the mutualism. As a corollary, warming also reduced density‐dependent processes among conspecific bees competing for floral resources, as greater bee abundance and greater numbers of flower visits increased flower handling time.

Warmed flowers had lower nectar volume, likely driven by a combination of increased evaporation of the aqueous part of nectar and reduced water available for nectar production (McCombs et al. 2022). Despite species‐specific differences in water use that reflect physiological differences among the three plant species (de Manincor et al. 2023), physiological stress under warmer conditions likely constrained plant investment in floral tissues (Descamps et al. 2020; Teixido et al. 2016), resulting in smaller flowers. Thus, both directly and indirectly via reduced flower size, warming reduced per‐flower nectar available to bees, likely indirectly affecting their survival.

As a direct consequence of reduced nectar volume, bees visited more flowers (Figure 3A,G). At the same time, lower nectar volume directly increased flower handling time (Figure 3A). Therefore, bees would have needed to both make more visits and spend more time per visit to collect nectar rewards from warmed plants. This finding suggests that, although nectar volume and sugar concentration were inversely related, nectar rewards per flower were not equivalent across ambient vs. warmed communities. The chain of effects linking temperature, nectar volume, and handling time (Figure 3A) points to increasing energetic costs for bees in a warmer climate, with greater energy required to obtain nectar to sustain foraging and to provision offspring, as well as longer times spent away from nests (Gérard et al. 2024; McCallum et al. 2013).

The overall effect of warming on flower visitation was negative, operating through reductions in both bee and flower abundances and changes to floral traits. Climate warming is therefore expected to weaken this mutualistic interaction, as reduced interaction frequency translates into reduced exchange of resources (namely nectar and pollen) and services (pollen transfer), the currencies of plant–pollinator mutualisms.

### 4.3 Warming Depresses Plant Fitness

Direct, warming‐induced reductions in the number of seeds per fruit (Figure 4A,B) likely reflect constrained pollen tube growth and/or the development of ovules into seeds under high temperatures (Gray and Brady 2016; Rosenberger et al. 2024). Thus, although warming positively affected the number of ovules per flower (Figure 4A) and the numbers of ovules and seeds per fruit were positively related (Figure 4A,C), the probability that these additional ovules developed into seeds was lower, reducing seed set under warming (Figure 4A). It is also possible that warming impaired pollen development or viability, depressing seed set (Chaturvedi et al. 2021; Rosenberger et al. 2024). Because ovule and seed production under ambient conditions fell within the expected ranges for all three plant species (Andersson 1994; Armbruster et al. 2002; Jorgensen and Arathi 2013; Vasile et al. 2021), we can attribute diminished reproductive output in the warmed communities to warming‐induced stress.

Although the number of seeds per fruit was higher in ambient communities (Figure 4A,B), greater bee visit frequency had opposing effects on seed quality. Interestingly, seeds were less likely to germinate (Figure 4A,D), but seedlings were more likely to survive (Figure 4A,E). This suggests that more visits transferred more pollen to flowers, yielding more seeds per ovule, but the resulting seeds were of lower quality in terms of germinability. Competition for floral resources under ambient temperatures, as suggested by increased visits and handling times in the dynamic model, may have led to greater self‐pollination through intra‐flower or intra‐individual pollen flow, causing seeds to have lower viability (Kehrberger and Holzschuh 2019; Mitchell et al. 2009). At the same time, those seeds that were viable were superior in quality to those produced from less‐frequent bee visits in warmed communities, as evidenced by the lower mortality rates of seedlings originating from unwarmed communities.

By exposing parent plants to different warming scenarios and germinating their seeds under common garden conditions, we tested for parental effects of warming on seed germination and seedling mortality. We found a direct, positive parental effect of warming on the germination success of seeds (Figure 4A,F) and a larger direct increase in seedling mortality (Figure 4A,G). Beneficial parental effects on seed germination have been found for other species in warmed conditions (e.g., Gray and Brady 2016; Vasile et al. 2021). In our modelling approach, we can separate the direct and indirect effects of warming, the latter emerging via changes in abundances and plant–pollinator interaction strength. Considering all pathways by which temperature affected seed germination and seedling mortality, the effect of warming on germinability remained positive, whereas the overwhelming effect of warming on seedlings was to increase mortality.

Depressed plant fitness in a warmer world will likely have far‐reaching consequences for ecosystem functioning, as flowering plants form the foundation of terrestrial food webs (Lippmann et al. 2019; Mora et al. 2015; Pocock et al. 2012). Given a scenario wherein warmer temperatures cause plants to produce fewer seeds that are more likely to germinate but then quickly die prior to developing cotyledons, as we found, the consequence could be depletion of local seed banks (Dwyer and Erickson 2016). Warmer climates are thus likely to impose a net cost on plant fitness: plants may benefit from increased seed germination but suffer a greater cost through increased seedling mortality, potentially reducing plant population sizes. Therefore, rapid depletion of life stages critical for the persistence of species, such as the seed banks of annual plants, puts them at particular risk of local extinction.

### 4.4 Warming Depresses Bee Fitness

Warming reduced bee reproductive output by two metrics, the number of nest cells that bees provisioned and the number of offspring that survived to the cocoon‐spinning prepupal stage. Given nesting success under ambient conditions fell within the expected range for our focal bee species, *O. lignaria* (Bosch et al. 2000; Boyle and Pitts‐Singer 2019), depressed reproduction under warming signals warming‐induced stress.

As expected, greater reproductive effort corresponded to greater reproductive success as quantified by offspring surviving to the cocoon stage (Figure 5A,F). However, warming had a direct negative effect on the number of cocoons produced per community (Figure 5A,G), pointing to lethal effects of a warmer climate during the early stages of bee offspring development. Although other studies have found negative effects of extreme heat on larval development of *O. lignaria* (Melone et al. 2024; Walters et al. 2024), here we pinpoint underlying environmental drivers of reduced bee fitness and show that sustained warming of lower magnitudes can also induce larval mortality in this important pollinator and likely in other solitary bees, which do not behaviorally thermoregulate their nests.

In addition to the direct lethal effects of warming, bee offspring development can be impaired under conditions of nutritional stress. Nesting is energetically costly for solitary bees, and low‐quality floral resources can depress both egg laying and successful offspring maturation (Ostap‐Chec et al. 2021; Walters et al. 2024; Williams 2003). Although we measured only nectar production, changes in pollen quality are also likely in heat‐stressed plants (Borghi et al. 2019; Chaturvedi et al. 2021). Consumption of pollen from such plants can drastically reduce larval survival, increasing the likelihood of mortality before pupation (Walters et al. 2024). Overall, warming can negatively affect bee reproductive success both directly and indirectly, threatening the persistence of univoltine solitary bees.

Reinforcing the idea that bees in unwarmed environments experienced competition for floral resources, such as nectar, bees had longer handling times when flowers had smaller nectar volumes (Figure 5A,B). Thus, greater bee abundances indirectly increased handling time, suggesting density‐driven resource limitation in the absence of warming. These findings support the inference that depressed bee fitness in warmed communities reflects warming‐induced lethal and sublethal physiological stress, rather than arising as an artifact of experimental conditions. Altogether, our results indicate that reduced nectar availability, flower visitation, and bee survival under warming all contribute to lower bee reproductive success, further diminishing this mutualism.

## 5 Conclusions

By simultaneously subjecting entire experimental communities of plants and bees to different warming trajectories, we were able to isolate the effects of temperature while revealing emergent properties that arise only from analyzing the system as a whole. Although individual relationships between environmental stressors and traits can identify consequences for each species and their interactions (de Manincor et al. 2023; Pérez‐Ramos et al. 2019; Walters et al. 2024), they do not expose the complex direct and cascading effects shown by our analyses. Recent work on natural communities found indirect negative effects of warming on plant (Zhang et al. 2025) and solitary bee (Wong et al. 2025) fitness components via altered flowering phenology and floral density, respectively, similar to our findings. However, several potentially important variables and pathways were not included due to inherent challenges of field studies, and the fitness of both partners could not be considered simultaneously. Our approach informs our understanding of how a sustained stressor such as climatic warming plays out over time as a community of plants and pollinators progresses from pre‐flowering and pre‐emergence to senescence. Captured in these dynamics are the demographic, phenological, and physiological responses of each partner. By subjecting both plants and bees to the same late‐winter environmental conditions prior to the onset of flowering and emergence, we demonstrate that phenological mismatches are unlikely when temporal overlap between full phenological abundance curves is considered. We initiated warming 2.5 weeks before the end of winter and once plants and bees reached the same relative developmental point, prior to flowering and emergence. Thus, from a starting point of synchrony, we captured the effects of late‐winter and sustained spring warming, which allowed for shifts in flowering onset and bee emergence. Our findings draw attention away from concerns about climate change causing phenological mismatches to the physiological and behavioral changes that we show can erode the strength of plant–pollinator mutualisms. Thus, phenological synchrony alone is an unreliable indicator of the vulnerability of mutualisms to disruption.

Ecologists have long recognized the threat of interaction weakening and disruption preceding local population extinction under environmental change (Janzen 1974). Yet empirical evidence showing interactions weakening in real time and data on the trait‐based mechanisms from which interaction disruption emerges have been elusive. In this study, we have shown not only that warming weakens the mutualism between plants and pollinators but also that this weakening emerges from stress‐induced responses of both partners throughout their life cycles. Our work indicates that warming will have pervasive negative effects on the interactions that underpin plant and pollinator communities.

## Author Contributions

**Natasha de Manincor:** conceptualization, data curation, investigation, methodology, validation, writing – original draft, writing – review and editing. **Alessandro Fisogni:** data curation, formal analysis, investigation, methodology, validation, visualization, writing – original draft, writing – review and editing. **Oscar Godoy:** formal analysis, writing – review and editing. **Nicole E. Rafferty:** conceptualization, data curation, funding acquisition, investigation, methodology, project administration, supervision, validation, writing – original draft, writing – review and editing.

## Funding

This work was supported by the Hellman Fellows Program and USDA National Institute of Food and Agriculture (1026313).

## Conflicts of Interest

The authors declare no conflicts of interest.

## Supporting information


**Figure S1:** Correlations between (A) plant and (B) pollinator phenological distribution means and temperature. Solid lines indicate significant interactions between plant and pollinator regressions (model coefficients in Table S5). Points represent values recorded in the 20 foraging arenas over the duration of the experiment, and shaded areas represent 95% CIs.
**Figure S2:** Correlation between temperature and volumetric water content (% VWC) measured in plant soil. Points represent values recorded for the three plant species over the duration of the experiment, and shaded areas represent 95% CIs. CH: *Collinsia heterophylla* , NM: *Nemophila menziesii* , PC: *Phacelia campanularia* .
**Table S1:** (A) Temperature setpoints for the ambient treatment throughout the experiment and (B, C) model coefficients obtained from repeated measurements ANOVAs to evaluate temporal differences between the four temperature treatments throughout the experiment. (B) Estimated average temperature increase in three warming scenarios compared to ambient temperature (intercept) in Riverside, California, USA, based on historic monthly climate normals for 1991–2020; (C) pairwise comparisons between the four temperature treatments (estimated marginal means and Tukey‐adjusted *p*‐values).
**Table S2:** Hypotheses underlying the causal paths included in the dynamic (D), plant fitness (P), and bee fitness (B) piecewise structural equation models.
**Table S3:** Linear paths (GLMMs) included in the dynamic (D), plant fitness (P), and bee fitness (B) piecewise structural equation models (pSEM). Day of year was included as random factor only in the dynamic pSEM. Number of flower visits and flower handling time were log‐transformed to approximate normality of the residuals.
**Table S4:** Non‐causal error correlations included in the dynamic (D), plant fitness (P), and bee fitness (B) piecewise structural equation models.
**Table S5:** Model coefficients obtained from linear models to evaluate interaction effects between estimators of flowering and pollinator (bee) phenological distributions and increasing temperature. Bee distributions are contrasted against flower distributions in all models. Temperatures were estimated as means at the foraging arena level over the duration of the experiment.
**Table S6:** Model coefficients obtained from GLMMs to evaluate shifts of flowering and pollinator phenology in relation to temperature throughout the duration of the experiment. Quadratic terms for day of year (DOY) were included to allow for non‐linear effects. Temperatures were estimated as daily means at the arena level.
**Table S7:** Model coefficients obtained from a beta regression to evaluate relationships between plant–pollinator phenology overlap and increasing temperatures. Temperatures were estimated as means at the foraging arena level over the duration of the experiment, and a squared effect was included to account for non‐linear relationships.
**Table S8:** Unstandardized and standardized path coefficients obtained from the dynamic pSEM (Figure 3). Fisher's *C* value = 2.49, *p* = 0.29, indicating good model fit.
**Table S9:** Model coefficients obtained from beta regressions to evaluate relationships between temperature and soil moisture (% volumetric water content), at the plant community level and in each plant species separately. Day of year was included as a random effect in all models to account for temporal autocorrelation, and plant species ID was included in the overall model to account for variability among species.
**Table S10:** Unstandardized and standardized path coefficients obtained from the pSEM on plant fitness (Figure 4). Fisher's *C* value = 12.79, *p* = 0.54, indicating good model fit.
**Table S11:** Unstandardized and standardized path coefficients obtained from the pSEM on bee fitness (Figure 5). Fisher's *C* value = 23.7, *p* = 0.25, indicating good model fit.

## Data Availability

The data that support the findings of this study are openly available in Zenodo at https://doi.org/10.5281/zenodo.15683270.
