# Supplementary material for: Climate Warming Drives the Breakdown of Plant–Pollinator Mutualisms Despite Phenological Synchrony
Source: Glob Chang Biol. 2026 Aug 26;32(8):e71074. doi: 10.1111/gcb.71074 (PMC13508041; doi:10.1111/gcb.71074)
Supplement: Supplementary file 1 [file GCB-32-e71074-s001.docx]

Supplementary information to ***“Climate warming drives the breakdown of plant-pollinator mutualisms despite phenological synchrony”*** by de Manincor et al.

**Running title:** The breakdown of plant-pollinator mutualisms

**List of Authors:** Natasha de Manincor^a1^*†, Alessandro Fisogni^a1^†, Oscar Godoy^b^, Nicole E. Rafferty^c^*

**List of Author’s ORCID #:**

Natasha de Manincor: 0000-0001-9696-125X

Alessandro Fisogni: 0000-0001-6179-2767

Oscar Godoy: 0000-0003-4988-6626

Nicole E. Rafferty: 0000-0002-6350-1705

**Joint First Authorship:** ^1^Natasha de Manincor and Alessandro Fisogni should be considered joint first author

**Institutional Affiliations:**

^a^ Department of Evolution, Ecology and Organismal Biology, University of California, Riverside; Riverside, CA, 92521, USA.

^b^ Estación Biológica de Doñana, Consejo Superior de Investigaciones Científicas (EBD-CSIC); Sevilla, 41092, Spain.

^c^ School of BioSciences, The University of Melbourne; Parkville, VIC, 3010, Australia.

† Present address: CEFE, CNRS, Univ Montpellier, EPHE, IRD, Montpellier, France.

**Corresponding Author Contact Information: ***Natasha de Manincor ([natasha.demanincor@gmail.com](mailto:natasha.demanincor@gmail.com)); *Nicole E. Rafferty ([nicole.rafferty@unimelb.edu.au](mailto:nicole.rafferty@unimelb.edu.au)**)**

**Figure S1** Correlations between (A) plant and (B) pollinator phenological distribution means and temperature. Solid lines indicate significant interactions between plant and pollinator regressions (model coefficients in Table S5). Points represent values recorded in the 20 foraging arenas over the duration of the experiment, and shaded areas represent 95% CIs.

**Figure S2** Correlation between temperature and volumetric water content (% VWC) measured in plant soil. Points represent values recorded for the three plant species over the duration of the experiment, and shaded areas represent 95% CIs. CH: *Collinsia heterophylla*, NM: *Nemophila menziesii*, PC: *Phacelia campanularia*.

**Table S1** (A) Temperature setpoints for the ambient treatment throughout the experiment and (B-C) model coefficients obtained from repeated measurements ANOVAs to evaluate temporal differences between the four temperature treatments throughout the experiment. (B) Estimated average temperature increase in three warming scenarios compared to ambient temperature (intercept) in Riverside, California, USA, based on historic monthly climate normals for 1991-2020; (C) pairwise comparisons between the four temperature treatments (estimated marginal means and Tukey-adjusted p-values).

(A)

| **Month** | **Day temperature (**℃) | **Night temperature (**℃) |
| --- | --- | --- |
| March | 21.5 | 7.0 |
| April | 23.3 | 7.7 |
| May | 26.2 | 10.4 |
| June | 30.1 | 12.6 |

(B)

| **Explanatory variables** | **Estimate** | **St. Error** | **df** | **t value** | ***P* value** |
| --- | --- | --- | --- | --- | --- |
| Intercept | 24.42 | 0.31 | 107.91 | 79.65 | < 2e^-16^ |
| Warming treatment 1 | 1.38 | 0.11 | 2631.8 | 12.63 | < 2e^-16^ |
| Warming treatment 2 | 2.48 | 0.11 | 2631.9 | 22.77 | < 2e^-16^ |
| Warming treatment 3 | 6.03 | 0.11 | 2633.4 | 55.30 | < 2e^-16^ |

(C)

| **Contrast** | **Estimate** | **St. Error** | **df** | **t ratio** | ***P* value** |
| --- | --- | --- | --- | --- | --- |
| Ambient – Warming 1 | −1.38 | 0.11 | 2632 | −12.63 | < 0.0001 |
| Ambient – Warming 2 | −2.48 | 0.11 | 2632 | −22.77 | < 0.0001 |
| Ambient – Warming 3 | −6.03 | 0.11 | 2634 | −55.30 | < 0.0001 |
| Warming 1 – Warming 2 | −1.10 | 0.11 | 2632 | −10.13 | < 0.0001 |
| Warming 1 – Warming 3 | −4.65 | 0.11 | 2634 | −42.69 | < 0.0001 |
| Warming 2 – Warming 3 | −3.55 | 0.11 | 2633 | −32.64 | < 0.0001 |

**Table S2** Hypotheses underlying the causal paths included in the dynamic (D), plant fitness (P), and bee fitness (B) piecewise structural equation models.

| Path | Model type | Hypotheses / alternate hypotheses | References |
| --- | --- | --- | --- |
| Warming → Number of bees | D; P; B | Sustained warming increases bee mortality / No effect of warming on number of bees | (Bosch et al., 2010; Kazenel et al., 2024; Vilchez-Russell & Rafferty, 2024) |
| Warming → Number of flowers | D; P; B | Warming depresses flower production / Warming stimulates flower production / No effect of warming on flower production | (de Manincor et al., 2023; Kuppler & Kotowska, 2021; McCombs et al., 2022; Moss & Evans, 2022; Zi et al., 2023) |
| Warming → Flower size | D | Warming reduces flower size / No effect of warming on flower size | (de Manincor et al., 2023; Descamps et al., 2020; Teixido et al., 2016) |
| Warming → Nectar volume | D; B | Warming reduces nectar volume / No effect of warming on nectar volume | (de Manincor et al., 2023; Descamps et al., 2020; McCombs et al., 2022; Moss & Evans, 2022) |
| Warming → Number of ovules | P | Warming reduces ovule production / Warming stimulates ovule production / No effect of warming on ovule production | (Chandler & Travers, 2024; Gray & Brady, 2016) |
| Warming → Number of seeds | P | Warming depresses seed production / Warming stimulates seed production / No effect of warming on seed production | (Gray & Brady, 2016; Rosenberger et al., 2024; Zi et al., 2023) |
| Warming → Seed mass | P | Warming negatively influences plant physiology and reduces seed mass / No effect of warming on seed mass | (de Manincor et al., 2023; Notarnicola et al., 2023; Zi et al., 2023) |
| Warming → Seed germination | P | Warming negatively influences parent plants and reduces seed germination / Warming positively influences parent plants and increases seed germination / No effect of warming on seed germination | (Dwyer & Erickson, 2016; Notarnicola et al., 2023; Whittle et al., 2009; Zhou & He, 2020) |
| Warming → Seedling mortality | P | Warming increases seedling mortality / Warming decreases seedling mortality / warming does not affect seedling mortality | (Chidumayo, 2008; Han et al., 2018; Milbau et al., 2017) |
| Warming → Number of cocoons | B | Warming negatively affects egg, larval and consequently cocoon development / Warming positively affects cocoon development / No effect of warming on cocoon development | (Kemp & Bosch, 2005; Melone et al., 2024; Ostap-Chec et al., 2021) |
| Number of bees → Number of flower visits | D; P; B | Fewer bees perform fewer visits / No effect of bee abundance on the number of visits | (Ebeling et al., 2008; Walters et al., 2024) |
| Number of bees → Flower handling time | D | Fewer bees spend more time foraging on flowers because of lower competition / No effect of bee abundance on flower foraging time | (Balfour et al., 2015) |
| Number of bees → Number of nest cells | B | More bees produce more nest cells until floral or nesting resources become limiting / No effect of bee abundance on nest cell production | (Stuligross & Williams, 2020) |
| Number of flowers → Number of flower visits | D; P; B | Fewer flowers increase competition for resources and reduce number of visits / Rewards are enough to guarantee same number of visits | (Benadi & Pauw, 2018; Ebeling et al., 2008; D. Sponsler et al., 2023) |
| Number of flowers → Flower handling time | D | Fewer flowers increase flower handling time because of reduced competition for pollinators / No effect of flower number on flower handling time | (Akter et al., 2017; Kehrberger & Holzschuh, 2019; Knauer et al., 2021) |
| Flower size → Nectar volume | D | Smaller flowers produce less nectar / Smaller flowers produce same amount of nectar | (Galetto & Bernardello, 2004; Parachnowitsch et al., 2019) |
| Flower size → Number of flower visits | D | Smaller flowers have lower attractiveness and are less visited by bees / Smaller flowers have same attractiveness to bees | (Delgado et al., 2023; Glaettli & Barrett, 2008; Reverté et al., 2023; Spaethe et al., 2001) |
| Flower size → Flower handling time | D | Smaller flowers are more difficult to handle and increase time spent on flowers by bees / Smaller flowers produce less nectar and reduce time spent on flowers by bees | (Harder, 1983; Reverté et al., 2023) |
| Flower size → Number of ovules | P | Smaller flowers produce fewer ovules / Smaller flowers produce same number of ovules | (Bawa et al., 2019; López et al., 1999; Mochizuki et al., 2019; Nepal et al., 2023) |
| Nectar volume → Number of flower visits | D; B | Lower nectar production reduces flower attractiveness and reduces number of visits / Lower nectar production does not reduce number of visits | (Fisogni et al., 2011; Pyke et al., 2020; Zhao et al., 2016) |
| Nectar volume → Flower handling time | D; B | Lower nectar production reduces bee foraging time / Lower nectar production does not affect foraging time | (Knauer et al., 2021; Li et al., 2021) |
| Number of flower visits → Flower handling time | D; B | Fewer visits decrease competition for floral resources and increase time spent foraging on flowers / More visits increase competition for floral resources and reduce time spent foraging on flowers | (Balfour et al., 2015; D. Sponsler et al., 2023; D. B. Sponsler et al., 2024) |
| Number of flower visits → Number of seeds | P | Fewer visits reduce conspecific pollen flow / More visits increase conspecific pollen flow / More visits reduce seed production through flower damage, stigma clogging or pollen tube competition / No effect of visits on seed production | (Herbertsson et al., 2021; Höfer et al., 2023; Lázaro et al., 2013; Morris et al., 2010; Ohara & Higashi, 1994; Russo et al., 2017) |
| Number of flower visits → Seed mass | P | Fewer visits reduce conspecific pollen flow / More visits increase conspecific pollen flow / No effect of visits on seed mass | (Bommarco et al., 2012; Fairhurst et al., 2022; Höfer et al., 2023) |
| Number of flower visits → Seed germination | P | Fewer visits reduce conspecific pollen flow / More visits increase conspecific pollen flow / No effect of visits on seed germination | (Christopher et al., 2021; Sasidharan et al., 2024) |
| Number of flower visits → Seedling mortality | P | Fewer visits reduce conspecific pollen flow / More visits increase conspecific pollen flow / No effect of visits on seedling mortality | (Dudash & Fenster, 2000; Schemske, 1983; Yates et al., 2007) |
| Number of flower visits → Number of nest cells | B | Fewer visits reduce energy intake in adults and pollen collection for larval development / More visits increase energy intake in adults and pollen collection for larval development / No effect of number of visits on nest building | (Bosch & Kemp, 2001; Goulson et al., 2015; Kim, 1999; Neff, 2008; Stuligross & Williams, 2020) |
| Number of flower visits → Number of cocoons | B | Fewer visits reduce pollen collection for larval development / More visits increase pollen collection for larval development / No effect of number of visits on larval development | (Bosch & Kemp, 2001; Goulson et al., 2015; Kim, 1999; Neff, 2008; Stuligross & Williams, 2020) |
| Flower handling time → Number of seeds | P | Longer visits enhance seed set because of higher pollen deposition / Longer visits reduce seed set because of increased self-pollen deposition / No effect of flower handling time on seed set | (King et al., 2013; Lienert & Fischer, 2004; Ohara & Higashi, 1994; Paul et al., 2024; Russo et al., 2017) |
| Flower handling time → Seed mass | P | Longer handling time increases probability of self-pollen deposition or stigma clogging, decreasing seed mass / Reduced handling decreases pollen deposition, decreasing seed mass / No effects of handling time on seed mass | (Fairhurst et al., 2022; Hirayama et al., 2005; Lienert & Fischer, 2004; Walsh et al., 2014) |
| Flower handling time → Seed germination | P | Longer handling time increases probability of self-pollen deposition, decreasing seed germination success / Reduced handling decreases pollen deposition, decreasing seed germination success / No effects of handling time on seed germination success | (Chautá-Mellizo et al., 2012; King et al., 2013; Lienert & Fischer, 2004; Paul et al., 2024) |
| Flower handling time → Number of nest cells | B | Shorter visits reduce energy intake in adults and pollen collection for larval development / Longer visits increase energy intake in adults and pollen collection for larval development / No effect of visit length on nest building | (Essenberg, 2013; Harder, 1983) |
| Flower handling time → Number of cocoons | B | Shorter visits reduce pollen collection for larval development / Longer visits increase pollen collection for larval development / No effect of visit length on larval development | (Essenberg, 2013; Harder, 1983) |
| Number of ovules → Number of seeds | P | Seed production is positively correlated with ovule availability / No relationship between number of ovules available and seed production | (Bawa et al., 2019; Ida et al., 2015; Strelin & Aizen, 2018) |
| Number of seeds → Seed mass | P | Trade-off between number of seeds produced and seed mass / No correlation between number of seeds produced and seed mass | (Leishman, 2001; Notarnicola et al., 2023; Tremayne & Richards, 2000) |
| Number of seeds → Seed germination | P | Indirect effect of number of seeds on seed germination success through seed mass, if trade-off between the two variables exists / No effect of number of seeds on seed germination success | (Leishman, 2001; Notarnicola et al., 2023; Tremayne & Richards, 2000) |
| Number of seeds → Seedling mortality | P | Trade-off between number of seeds produced and seed viability / No effect of number of seeds produced on seed viability | (Leishman, 2001; Muscarella et al., 2013) |
| Seed mass → Seed germination | P | Larger seeds have higher germination success than smaller seeds / Smaller seeds have higher germination success than larger seeds / No effect of seed mass on seed germination success | (Kahmen & Poschlod, 2008; Notarnicola et al., 2023; Sercu et al., 2021) |
| Seed germination → Seedling mortality | P | Higher germination increases the number of surviving seedlings / No correlation between seed germination and seedling mortality | (Larson et al., 2015; Moles & Westoby, 2004; Sales et al., 2013) |
| Number of nest cells → Number of cocoons | B | Number of nest cells is positively correlated with number of cocoons / No effect of number of nest cells on number of cocoons | (Bosch & Kemp, 2001; Spendal & Cane, 2022; Wong & Forrest, 2021) |

**Table S3** Linear paths (GLMMs) included in the dynamic (D), plant fitness (P), and bee fitness (B) piecewise structural equation models (pSEM). Day of year was included as random factor only in the dynamic pSEM. Number of flower visits and flower handling time were log-transformed to approximate normality of the residuals.

| **Response variable** | **Explanatory variables** | **Random effects** | **Error family** | **Model type** |
| --- | --- | --- | --- | --- |
| Number of bees | Temperature | Day of year, Arena identity | Poisson (log-link) | D; P; B |
| Number of flowers | Temperature | Day of year, Plant species identity, Arena identity | Poisson (log-link) | D; P; B |
| Flower size | Temperature | Day of year, Plant species identity, Arena identity | Gaussian (identity-link) | D |
| Nectar volume | Temperature, flower size | Day of year, Plant species identity, Arena identity | Gaussian (identity-link) | D; B |
| Number of flower visits | Number of bees, number of flowers, nectar volume | Day of year, Plant species identity, Arena identity | Gaussian (identity-link) | D; P; B |
| Flower handling time | Number of bees, number of flowers, flower size, number of visits, nectar volume | Day of year, Plant species identity, Arena identity | Gaussian (identity-link) | D; B |
| Number of ovules | Temperature, flower size | Plant species identity | Poisson (log-link) | P |
| Number of seeds | Temperature, number of ovules, number of flower visits, flower handling time | Plant species identity | Poisson (log-link) | P |
| Seed mass | Temperature, number of seeds, number of flower visits, flower handling time | Plant species identity | Gaussian (identity-link) | P |
| Seed germination | Temperature, number of seeds, seed mass, number of flower visits, flower handling time | Arena identity | Poisson (log-link) | P |
| Seedling mortality | Temperature, number of seeds, seed mass, seed germination, number of flower visits, flower handling time | Arena identity | Poisson (log-link) | P |
| Number of nest cells | Number of bees, number of flower visits, flower handling time | Arena identity | Poisson (log-link) | B |
| Number of cocoons | Temperature, number of flower visits, flower handling time, number of nest cells | Arena identity | Poisson (log-link) | B |

**Table S4** Non-causal error correlations included in the dynamic (D), plant fitness (P), and bee fitness (B) piecewise structural equation models.

| **Correlated errors** | **Model type** |
| --- | --- |
| Temperature ~ number of visits | D |
| Temperature ~ flower handling time | D |
| Bee abundance ~ flower size | D |
| Bee abundance ~ number of flowers | D; P; B |
| Bee abundance ~ number of ovules | P |
| Bee abundance ~ number of seeds | P |
| Flower handling time ~ flower size | D |
| Number of flowers ~ number of ovules | P |
| Number of flowers ~ number of seeds | P |
| Number of visits ~ number of ovules | P |
| Number of nest cells ~ number of flowers | B |
| Number of nest cells ~ nectar volume | B |

**Table S5** Model coefficients obtained from linear models to evaluate interaction effects between estimators of flowering and pollinator (bees) phenological distributions and increasing temperature. Bee distributions are contrasted against flower distributions in all models. Temperatures were estimated as means at the foraging arena level over the duration of the experiment.

| **Response variables** | **Explanatory variables** | **Estimate** | **St. Error** | **t value** | ***P* value** |
| --- | --- | --- | --- | --- | --- |
| Mean |  |  |  |  |  |
|  | Intercept | 340.54 | 56.02 | 6.08 | 5.46e^-07^ |
|  | Temperature | −6.24 | 2.06 | −3.03 | 4.51e^-03^ |
|  | Bees | −336.74 | 79.23 | −4.25 | 1.44e^-04^ |
|  | Bees:temperature | 6.144 | 2.91 | 2.11 | 0.04 |
| Skewness |  |  |  |  |  |
|  | Intercept | 2.30 | 0.81 | 2.85 | 7.11e^-03^ |
|  | Temperature | −0.07 | 0.03 | −2.30 | 0.03 |
|  | Bees | −2.72 | 1.14 | −2.38 | 0.02 |
|  | Bees:temperature | 0.11 | 0.04 | 2.69 | 0.01 |
| Breadth |  |  |  |  |  |
|  | Intercept | 2.33 | 1.75 | 1.33 | 0.19 |
|  | Temperature | −0.01 | 0.06 | −0.22 | 0.83 |
|  | Bees | −0.34 | 2.47 | −0.14 | 0.89 |
|  | Bees:temperature | 0.04 | 0.09 | 0.42 | 0.68 |

**Table S6** Model coefficients obtained from GLMMs to evaluate shifts of flowering and pollinator phenology in relation to temperature throughout the duration of the experiment. Quadratic terms for day of year (DOY) were included to allow for non-linear effects. Temperatures were estimated as daily means at the arena level.

| **Response variables** | **Explanatory variables** | **Estimate** | **St. Error** | **z value** | ***P* value** |
| --- | --- | --- | --- | --- | --- |
| Flowering phenology |  |  |  |  |  |
|  | Intercept | −4.14 | 0.845 | −4.95 | 7.55e−07 |
|  | Temperature | 0.19 | 0.03 | 5.88 | 4.13e−09 |
|  | DOY | 18.47 | 1.57 | 11.73 | < 2e−16 |
|  | DOY^2^ | 8.31 | 0.76 | 10.90 | < 2e−16 |
|  | Temperature:DOY | −0.34 | 0.06 | −5.83 | 5.50e−09 |
|  | Temperature:DOY^2^ | −0.26 | 0.03 | −9.23 | < 2e−16 |
| Pollinator phenology |  |  |  |  |  |
|  | Intercept | −3.50 | 1.21 | −2.90 | 0.004 |
|  | Temperature | 0.12 | 0.05 | 2.41 | 0.02 |
|  | DOY | 14.01 | 4.31 | 3.25 | 0.001 |
|  | DOY^2^ | 3.49 | 5.27 | 0.66 | 0.51 |
|  | Temperature:DOY | −0.37 | 0.16 | −2.23 | 0.03 |
|  | Temperature:DOY^2^ | −0.42 | 0.19 | −2.16 | 0.03 |

**Table S7** Model coefficients obtained from a beta regression to evaluate relationships between plant-pollinator phenology overlap and increasing temperatures. Temperatures were estimated as means at the foraging arena level over the duration of the experiment, and a squared effect was included to account for non-linear relationships.

| **Explanatory variables** | **Estimate** | **St. Error** | **t value** | ***P* value** |
| --- | --- | --- | --- | --- |
| Intercept | 14.85 | 9.47 | 1.57 | 0.12 |
| Temperature | −1.17 | 0.70 | −1.67 | 0.10 |
| Temperature squared | 0.02 | 0.01 | 1.67 | 0.09 |

**Table S8** Unstandardized and standardized path coefficients obtained from the dynamic pSEM (Figure 3). Fisher’s C value = 2.49, *P* = 0.29, indicating good model fit.

| **Response variables** | **Explanatory variables** | **Estimate** | **Std. Error** | **Std. Estimate** | ***P* value** |
| --- | --- | --- | --- | --- | --- |
| Flower abundance | Temperature | −0.044 | 0.001 | −0.069 | < 0.001 |
| Bee abundance | Temperature | −0.051 | 0.006 | −0.183 | < 0.001 |
| Flower size | Temperature | −0.027 | 0.0009 | −0.507 | < 0.001 |
| Nectar volume | Temperature | −0.049 | 0.003 | −0.197 | < 0.001 |
| Nectar volume | Flower size | 0.933 | 0.051 | 0.204 | < 0.001 |
| Number of flower visits | Bee abundance | 0.379 | 0.018 | 0.550 | < 0.001 |
| Number of flower visits | Flower abundance | 0.001 | 0.0002 | 0.123 | < 0.001 |
| Number of flower visits | Flower size | 0.116 | 0.123 | 0.024 | 0.345 |
| Number of flower visits | Nectar volume | −0.373 | 0.044 | −0.346 | < 0.001 |
| Flower handling time | Bee abundance | 0.067 | 0.010 | 0.118 | < 0.001 |
| Flower handling time | Flower abundance | 0.0001 | 0.0001 | 0.008 | 0.582 |
| Flower handling time | Number of flower visits | 0.608 | 0.011 | 0.739 | < 0.001 |
| Flower handling time | Flower size | −0.099 | 0.073 | −0.024 | 0.175 |
| Flower handling time | Nectar volume | −0.071 | 0.023 | −0.080 | 0.003 |

**Table S9** Model coefficients obtained from beta regressions to evaluate relationships between temperature and soil moisture (% volumetric water content), at the plant community level and in each plant species separately. Day of year was included as a random effect in all models to account for temporal autocorrelation, and plant species ID was included in the overall model to account for variability among species.

| **Model** | **Explanatory variables** | **Estimate** | **St. Error** | **z value** | ***P* value** |
| --- | --- | --- | --- | --- | --- |
| All plant species | Intercept | 0.14 | 0.78 | 0.18 | 0.86 |
|  | Temperature | −0.07 | 0.03 | −2.37 | 0.02 |
| *Collinsia heterophylla* | Intercept | −0.50 | 1.14 | −0.44 | 0.66 |
|  | Temperature | −0.05 | 0.04 | −1.07 | 0.29 |
| *Nemophila menziesii* | Intercept | 1.72 | 1.24 | 1.38 | 0.17 |
|  | Temperature | −0.12 | 0.05 | −2.47 | 0.01 |
| *Phacelia campanularia* | Intercept | −1.06 | 1.21 | −0.87 | 0.38 |
|  | Temperature | −0.05 | 0.05 | −0.95 | 0.34 |

**Table S10** Unstandardized and standardized path coefficients obtained from the pSEM on plant fitness (Figure 4). Fisher’s C value = 12.79, *P* = 0.54, indicating good model fit.

| **Response variables** | **Explanatory variables** | **Estimate** | **Std. Error** | **Std. Estimate** | ***P* value** |
| --- | --- | --- | --- | --- | --- |
| Flower abundance | Temperature | −0.032 | 0.003 | −0.146 | < 0.001 |
| Bee abundance | Temperature | −0.050 | 0.011 | −0.573 | < 0.001 |
| Number of ovules | Temperature | 0.021 | 0.004 | 0.085 | < 0.001 |
| Number of seeds | Temperature | −0.036 | 0.005 | −0.128 | < 0.001 |
| Seed germination | Temperature | 0.114 | 0.019 | 0.420 | < 0.001 |
| Seedling mortality | Temperature | 0.680 | 0.091 | 0.846 | < 0.001 |
| Number of flower visits | Bee abundance | 0.115 | 0.019 | 0.336 | < 0.001 |
| Number of flower visits | Flower abundance | −0.007 | 0.002 | −0.425 | < 0.001 |
| Number of seeds | Number of ovules | 0.022 | 0.001 | 0.753 | < 0.001 |
| Number of seeds | Number of visits | 0.104 | 0.027 | 0.078 | < 0.001 |
| Seed germination | Number of flower visits | −0.400 | 0.091 | −0.314 | < 0.001 |
| Seed germination | Number of seeds | 0.004 | 0.002 | 0.121 | 0.068 |
| Seedling mortality | Number of flower visits | −0.599 | 0.253 | −0.159 | 0.018 |
| Seedling mortality | Number of seeds | 0.008 | 0.005 | 0.097 | 0.115 |
| Seedling mortality | Seed germination | −0.077 | 0.130 | −0.049 | 0.552 |

**Table S11** Unstandardized and standardized path coefficients obtained from the pSEM on bee fitness (Figure 5). Fisher’s C value = 23.7, *P* = 0.25, indicating good model fit.

| **Response variables** | **Explanatory variables** | **Estimate** | **Std. Error** | **Std. Estimate** | ***P* value** |
| --- | --- | --- | --- | --- | --- |
| Flower abundance | Temperature | −0.032 | 0.003 | −0.146 | < 0.001 |
| Bee abundance | Temperature | −0.050 | 0.011 | −0.573 | < 0.001 |
| Nectar volume | Temperature | −0.622 | 0.079 | −0.274 | < 0.001 |
| Number of cocoons | Temperature | −0.958 | 0.274 | −0.097 | < 0.001 |
| Number of flower visits | Bee abundance | 0.141 | 0.020 | 0.413 | < 0.001 |
| Number of flower visits | Flower abundance | −0.006 | 0.002 | −0.407 | < 0.001 |
| Number of flower visits | Nectar volume | −0.040 | 0.009 | −0.427 | < 0.001 |
| Flower handling time | Nectar volume | −0.014 | 0.005 | −0.244 | 0.007 |
| Flower handling time | Number of flower visits | 0.327 | 0.038 | 0.523 | < 0.001 |
| Number of nest cells | Bee abundance | 0.423 | 0.016 | 0.630 | < 0.001 |
| Number of nest cells | Number of flower visits | 0.434 | 0.053 | 0.221 | < 0.001 |
| Number of nest cells | Flower handling time | −0.294 | 0.071 | −0.094 | < 0.001 |
| Number of cocoons | Number of flower visits | −0.013 | 0.132 | −0.0003 | 0.924 |
| Number of cocoons | Flower handling time | 0.004 | 0.176 | 0.0001 | 0.981 |
| Number of cocoons | Number of nest cells | 0.856 | 0.185 | 0.687 | < 0.001 |

**SI References**

Akter, A., Biella, P., & Klecka, J. (2017). Effects of small-scale clustering of flowers on pollinator foraging behaviour and flower visitation rate. *PLoS ONE*, *12*(11), e0187976. https://doi.org/10.1371/journal.pone.0187976

Balfour, N. J., Gandy, S., & Ratnieks, F. L. W. (2015). Exploitative competition alters bee foraging and flower choice. *Behavioral Ecology and Sociobiology*, *69*(10), 1731–1738. https://doi.org/10.1007/s00265-015-1985-y

Bawa, K. S., Ingty, T., Revell, L. J., & Shivaprakash, K. N. (2019). Correlated evolution of flower size and seed number in flowering plants (monocotyledons). *Annals of Botany*, *123*(1), 181–190. https://doi.org/10.1093/aob/mcy154

Benadi, G., & Pauw, A. (2018). Frequency dependence of pollinator visitation rates suggests that pollination niches can allow plant species coexistence. *Journal of Ecology*, *106*(5), 1892–1901. https://doi.org/10.1111/1365-2745.13025

Bommarco, R., Marini, L., & Vaissière, B. E. (2012). Insect pollination enhances seed yield, quality, and market value in oilseed rape. *Oecologia*, *169*(4), 1025–1032. https://doi.org/10.1007/s00442-012-2271-6

Bosch, J., & Kemp, W. P. (2001). *How to manage the blue orchard bee*. Sustainable Agriculture Network.

Bosch, J., Sgolastra, F., & Kemp, W. P. (2010). Timing of eclosion affects diapause development, fat body consumption and longevity in *Osmia lignaria*, a univoltine, adult-wintering solitary bee. *Journal of Insect Physiology*, *56*(12), 1949–1957. https://doi.org/10.1016/j.jinsphys.2010.08.017

Chandler, E. K., & Travers, S. E. (2024). Intraspecific variation in responses to extreme and moderate temperature stress in the wild species, *Solanum carolinense* (Solanaceae). *AoB PLANTS*, *16*(4), plae030. https://doi.org/10.1093/aobpla/plae030

Chautá-Mellizo, A., Campbell, S. A., Bonilla, M. A., Thaler, J. S., & Poveda, K. (2012). Effects of natural and artificial pollination on fruit and offspring quality. *Basic and Applied Ecology*, *13*(6), 524–532. https://doi.org/10.1016/j.baae.2012.08.013

Chidumayo, E. N. (2008). Implications of climate warming on seedling emergence and mortality of African savanna woody plants. *Plant Ecology*, *198*(1), 61–71. https://doi.org/10.1007/s11258-007-9385-7

Christopher, D. A., Karron, J. D., Semski, W. R., Smallwood, P. A., Trapnell, D. W., & Mitchell, R. J. (2021). Selfing rates vary with floral display, pollinator visitation and plant density in natural populations of *Mimulus ringens*. *Journal of Evolutionary Biology*, *34*(5), 803–815. https://doi.org/10.1111/jeb.13781

de Manincor, N., Fisogni, A., & Rafferty, N. E. (2023). Warming of experimental plant–pollinator communities advances phenologies, alters traits, reduces interactions and depresses reproduction. *Ecology Letters*, *26*(2), 323–334. https://doi.org/10.1111/ele.14158

Delgado, T., Leal, L. C., El Ottra, J. H. L., Brito, V. L. G., & Nogueira, A. (2023). Flower size affects bee species visitation pattern on flowers with poricidal anthers across pollination studies. *Flora*, *299*, 152198. hsttp://doi.org/10.1016/j.flora.2022.152198

Descamps, C., Marée, S., Hugon, S., Quinet, M., & Jacquemart, A.-L. (2020). Species-specific responses to combined water stress and increasing temperatures in two bee-pollinated congeners (*Echium*, Boraginaceae). *Ecology and Evolution*, *10*(13), 6549–6561. https://doi.org/10.1002/ece3.6389

Dudash, M. R., & Fenster, C. B. (2000). Inbreeding and outbreeding depression in fragmented populations. In A. G. Young & G. M. Clarke (Eds.), *Genetics, Demography and Viability of Fragmented Populations* (pp. 35–54). Cambridge University Press. https://doi.org/10.1017/CBO9780511623448.005

Dwyer, J. M., & Erickson, T. E. (2016). Warmer seed environments increase germination fractions in Australian winter annual plant species. *Ecosphere*, *7*(10), e01497. https://doi.org/10.1002/ecs2.1497

Ebeling, A., Klein, A.-M., Schumacher, J., Weisser, W. W., & Tscharntke, T. (2008). How does plant richness affect pollinator richness and temporal stability of flower visits? *Oikos*, *117*(12), 1808–1815. https://doi.org/10.1111/j.1600-0706.2008.16819.x

Essenberg, C. J. (2013). Explaining the effects of floral density on flower visitor species composition. *The American Naturalist*, *181*(3), 344–356. https://doi.org/10.1086/669157

Fairhurst, S. M., Jackson, G. E., Evans, A., & Cole, L. J. (2022). The effect of pollination on the growth and reproduction of oilseed rape (*Brassica napus*). *Basic and Applied Ecology*, *63*, 164–174. https://doi.org/10.1016/j.baae.2022.06.007

Fisogni, A., Cristofolini, G., Rossi, M., & Galloni, M. (2011). Pollinator directionality as a response to nectar gradient: Promoting outcrossing while avoiding geitonogamy. *Plant Biology*, *13*(6), 848–856. https://doi.org/10.1111/j.1438-8677.2011.00453.x

Galetto, L., & Bernardello, G. (2004). Floral nectaries, nectar production dynamics and chemical composition in six *Ipomoea* species (Convolvulaceae) in relation to pollinators. *Annals of Botany*, *94*(2), 269–280. https://doi.org/10.1093/aob/mch137

Glaettli, M., & Barrett, S. C. H. (2008). Pollinator responses to variation in floral display and flower size in dioecious *Sagittaria latifolia* (Alismataceae). *New Phytologist*, *179*(4), 1193–1201. https://doi.org/10.1111/j.1469-8137.2008.02532.x

Goulson, D., Nicholls, E., Botías, C., & Rotheray, E. L. (2015). Bee declines driven by combined stress from parasites, pesticides, and lack of flowers. *Science*, *347*(6229), 1255957. https://doi.org/10.1126/science.1255957

Gray, S. B., & Brady, S. M. (2016). Plant developmental responses to climate change. *Developmental Biology*, *419*(1), 64–77. https://doi.org/10.1016/j.ydbio.2016.07.023

Han, A. R., Kim, H. J., Jung, J. B., & Park, P. S. (2018). Seed germination and initial seedling survival of the subalpine tree species, *Picea jezoensis,* on different forest floor substrates under elevated temperature. *Forest Ecology and Management*, *429*, 579–588. https://doi.org/10.1016/j.foreco.2018.07.042

Harder, L. D. (1983). Flower handling efficiency of bumble bees: Morphological aspects of probing time. *Oecologia*, *57*(1), 274–280. https://doi.org/10.1007/BF00379591

Herbertsson, L., Ekroos, J., Albrecht, M., Bartomeus, I., Batáry, P., Bommarco, R., Caplat, P., Diekötter, T., Eikestam, J., Entling, M. H., Farbu, S., Farwig, N., Gonzalez-Varo, J., Hass, A., Holzschuh, A., Hopfenmüller, S., Jakobsson, A., Jauker, B., Kovács-Hostyánszki, A., … Smith, H. (2021). Bees increase seed set of wild plants while the proportion of arable land has a variable effect on pollination in European agricultural landscapes. *Plant Ecology and Evolution*, *154*(3), Article 3. https://doi.org/10.5091/plecevo.2021.1884

Hirayama, K., Ishida, K., & Tomaru, N. (2005). Effects of pollen shortage and self-pollination on seed production of an endangered tree, *Magnolia stellata*. *Annals of Botany*, *95*(6), 1009–1015. https://doi.org/10.1093/aob/mci107

Höfer, R. J., Lindner, T., Ayasse, M., & Kuppler, J. (2023). Reduced seed set under water deficit is driven mainly by reduced flower numbers and not by changes in flower visitations and pollination. *Functional Ecology*, *37*(2), 461–471. https://doi.org/10.1111/1365-2435.14233

Ida, T. Y., Harder, L. D., & Kudo, G. (2015). The consequences of demand-driven seed provisioning for sexual differences in reproductive investment in *Thalictrum occidentale* (Ranunculaceae). *Journal of Ecology*, *103*(1), 269–280. https://doi.org/10.1111/1365-2745.12330

Kahmen, S., & Poschlod, P. (2008). Does germination success differ with respect to seed mass and germination season? Experimental testing of plant functional trait responses to grassland management. *Annals of Botany*, *101*(4), 541–548. https://doi.org/10.1093/aob/mcm311

Kazenel, M. R., Wright, K. W., Griswold, T., Whitney, K. D., & Rudgers, J. A. (2024). Heat and desiccation tolerances predict bee abundance under climate change. *Nature*, *628*(8007), 342–348. https://doi.org/10.1038/s41586-024-07241-2

Kehrberger, S., & Holzschuh, A. (2019). How does timing of flowering affect competition for pollinators, flower visitation and seed set in an early spring grassland plant? *Scientific Reports*, *9*(1), 15593. https://doi.org/10.1038/s41598-019-51916-0

Kemp, W. P., & Bosch, J. (2005). Effect of temperature on *Osmia lignaria* (Hymenoptera: Megachilidae) prepupa-adult development, survival, and emergence. *Journal of Economic Entomology*, *98*(6), 1917–1923. https://doi.org/10.1093/jee/98.6.1917

Kim, J. (1999). Influence of resource level on maternal investment in a leaf-cutter bee (Hymenoptera: Megachilidae). *Behavioral Ecology*, *10*(5), 552–556. https://doi.org/10.1093/beheco/10.5.552

King, C., Ballantyne, G., & Willmer, P. G. (2013). Why flower visitation is a poor proxy for pollination: Measuring single-visit pollen deposition, with implications for pollination networks and conservation. *Methods in Ecology and Evolution*, *4*(9), 811–818. https://doi.org/10.1111/2041-210X.12074

Knauer, A. C., Kokko, H., & Schiestl, F. P. (2021). Pollinator behaviour and resource limitation maintain honest floral signalling. *Functional Ecology*, *35*(11), 2536–2549. https://doi.org/10.1111/1365-2435.13905

Kuppler, J., & Kotowska, M. M. (2021). A meta-analysis of responses in floral traits and flower–visitor interactions to water deficit. *Global Change Biology*, *27*(13), 3095–3108. https://doi.org/10.1111/gcb.15621

Larson, J. E., Sheley, R. L., Hardegree, S. P., Doescher, P. S., & James, J. J. (2015). Seed and seedling traits affecting critical life stage transitions and recruitment outcomes in dryland grasses. *Journal of Applied Ecology*, *52*(1), 199–209. https://doi.org/10.1111/1365-2664.12350

Lázaro, A., Jakobsson, A., & Totland, Ø. (2013). How do pollinator visitation rate and seed set relate to species’ floral traits and community context? *Oecologia*, *173*(3), 881–893. https://doi.org/10.1007/s00442-013-2652-5

Leishman, M. R. (2001). Does the seed size/number trade-off model determine plant community structure? An assessment of the model mechanisms and their generality. *Oikos*, *93*(2), 294–302. https://doi.org/10.1034/j.1600-0706.2001.930212.x

Li, D., Yan, X., Lin, Y., Wang, L., & Wang, Q. (2021). Do flowers removed of either nectar or pollen attract fewer bumblebee pollinators? An experimental test in *Impatiens oxyanthera*. *AoB PLANTS*, *13*(4), plab029. https://doi.org/10.1093/aobpla/plab029

Lienert, J., & Fischer, M. (2004). Experimental inbreeding reduces seed production and germination independent of fragmentation of populations of *Swertia perennis*. *Basic and Applied Ecology*, *5*(1), 43–52. https://doi.org/10.1078/1439-1791-00212

López, J., Rodríguez-Riaño, T., Ortega-Olivencia, A., Antonio Devesa, J., & Ruiz, T. (1999). Pollination mechanisms and pollen-ovule ratios in some Genisteae (Fabaceae) from Southwestern Europe. *Plant Systematics and Evolution*, *216*(1), 23–47. https://doi.org/10.1007/BF00985099

McCombs, A. L., Debinski, D., Reinhardt, K., Germino, M. J., & Caragea, P. (2022). Warming temperatures affect meadow-wide nectar resources, with implications for plant–pollinator communities. *Ecosphere*, *13*(7), e4162. https://doi.org/10.1002/ecs2.4162

Melone, G. G., Stuligross, C., & Williams, N. M. (2024). Heatwaves increase larval mortality and delay development of a solitary bee. *Ecological Entomology*, *49*(3), 433–444. https://doi.org/10.1111/een.13317

Milbau, A., Vandeplas, N., Kockelbergh, F., & Nijs, I. (2017). Both seed germination and seedling mortality increase with experimental warming and fertilization in a subarctic tundra. *AoB PLANTS*, *9*(5), plx040. https://doi.org/10.1093/aobpla/plx040

Mochizuki, J., Itagaki, T., Aoyagi Blue, Y., Ito, M., & Sakai, S. (2019). Ovule and seed production patterns in relation to flower size variations in actinomorphic and zygomorphic flower species. *AoB PLANTS*, *11*(5), plz061. https://doi.org/10.1093/aobpla/plz061

Moles, A. T., & Westoby, M. (2004). What do seedlings die from and what are the implications for evolution of seed size? *Oikos*, *106*(1), 193–199. https://doi.org/10.1111/j.0030-1299.2004.13101.x

Morris, W. F., Vázquez, D. P., & Chacoff, N. P. (2010). Benefit and cost curves for typical pollination mutualisms. *Ecology*, *91*(5), 1276–1285. https://doi.org/10.1890/08-2278.1

Moss, E. D., & Evans, D. M. (2022). Experimental climate warming reduces floral resources and alters insect visitation and wildflower seed set in a cereal agro-ecosystem. *Frontiers in Plant Science*, *13*, 826205. https://doi.org/10.3389/fpls.2022.826205

Muscarella, R., Uriarte, M., Forero-Montaña, J., Comita, L. S., Swenson, N. G., Thompson, J., Nytch, C. J., Jonckheere, I., & Zimmerman, J. K. (2013). Life-history trade-offs during the seed-to-seedling transition in a subtropical wet forest community. *Journal of Ecology*, *101*(1), 171–182. https://doi.org/10.1111/1365-2745.12027

Neff, J. L. (2008). Components of nest provisioning behavior in solitary bees (Hymenoptera: Apoidea). *Apidologie*, *39*(1), 30–45. https://doi.org/10.1051/apido:2007055

Nepal, S., Trunschke, J., Ren, Z.-X., Burgess, K. S., & Wang, H. (2023). Community-wide patterns in pollen and ovule production, their ratio (P/O), and other floral traits along an elevation gradient in southwestern China. *BMC Plant Biology*, *23*(1), 425. https://doi.org/10.1186/s12870-023-04433-2

Notarnicola, R. F., Nicotra, A. B., Kruuk, L. E. B., & Arnold, P. A. (2023). Effects of warming temperatures on germination responses and trade-offs between seed traits in an alpine plant. *Journal of Ecology*, *111*(1), 62–76. https://doi.org/10.1111/1365-2745.14014

Ohara, M., & Higashi, S. (1994). Effects of inflorescence size on visits from pollinators and seed set of *Corydalis ambigua* (Papaveraceae). *Oecologia*, *98*(1), 25–30. https://doi.org/10.1007/BF00326086

Ostap-Chec, M., Kierat, J., Kuszewska, K., & Woyciechowski, M. (2021). Red mason bee (*Osmia bicornis*) thermal preferences for nest sites and their effects on offspring survival. *Apidologie*, *52*(3), 707–719. https://doi.org/10.1007/s13592-021-00858-6

Parachnowitsch, A. L., Manson, J. S., & Sletvold, N. (2019). Evolutionary ecology of nectar. *Annals of Botany*, *123*(2), 247–261. https://doi.org/10.1093/aob/mcy132

Paul, S., Roy, R., Singha, T., Debbarma, P., & Datta, B. K. (2024). Foraging behavior and pollination efficiency of generalist floral visitors of *Leucas aspera* (Willd.) link (Lamiaceae). *Journal of Asia-Pacific Biodiversity*, *17*(4), 644–652. https://doi.org/10.1016/j.japb.2024.04.015

Pyke, G. H., Kalman, J. R. M., Bordin, D. M., Blanes, L., & Doble, P. A. (2020). Patterns of floral nectar standing crops allow plants to manipulate their pollinators. *Scientific Reports*, *10*(1), 1660. https://doi.org/10.1038/s41598-020-58102-7

Reverté, S., Gérard, M., Bodson, M., Descamps, C., Gosselin, M., Jacquemart, A.-L., Louvieaux, J., Smagghe, G., Vandamme, P., Vereecken, N. J., & Michez, D. (2023). Intraspecific size shifts in generalist bumblebees and flowers lead to low functional consequences. *Ecosphere*, *14*(9), e4640. https://doi.org/10.1002/ecs2.4640

Rosenberger, N. M., Hemberger, J. A., & Williams, N. M. (2024). Heatwaves exacerbate pollen limitation through reductions in pollen production and pollen vigour. *AoB PLANTS*, *16*(5), plae045. https://doi.org/10.1093/aobpla/plae045

Russo, L., Park, M. G., Blitzer, E. J., & Danforth, B. N. (2017). Flower handling behavior and abundance determine the relative contribution of pollinators to seed set in apple orchards. *Agriculture, Ecosystems & Environment*, *246*, 102–108. https://doi.org/10.1016/j.agee.2017.05.033

Sales, N. M., Pérez-García, F., & Silveira, F. A. O. (2013). Consistent variation in seed germination across an environmental gradient in a Neotropical savanna. *South African Journal of Botany*, *87*, 129–133. https://doi.org/10.1016/j.sajb.2013.04.001

Sasidharan, R., Grond, S. G., Champion, S., Eilers, E. J., & Müller, C. (2024). Intraspecific plant chemodiversity at the individual and plot levels influences flower visitor groups with consequences for germination success. *Functional Ecology*, *38*(12), 2665–2678. https://doi.org/10.1111/1365-2435.14673

Schemske, D. W. (1983). Breeding system and habitat effects on fitness components in three neotropical *Costus* (Zingiberaceae). *Evolution*, *37*(3), 523–539. https://doi.org/10.1111/j.1558-5646.1983.tb05569.x

Sercu, B. K., Moeneclaey, I., Goeminne, B., Bonte, D., & Baeten, L. (2021). Flowering phenology and reproduction of a forest understorey plant species in response to the local environment. *Plant Ecology*, *222*(6), 749–760. https://doi.org/10.1007/s11258-020-01104-2

Spaethe, J., Tautz, J., & Chittka, L. (2001). Visual constraints in foraging bumblebees: Flower size and color affect search time and flight behavior. *Proceedings of the National Academy of Sciences*, *98*(7), 3898–3903. https://doi.org/10.1073/pnas.071053098

Spendal, R. C., & Cane, J. H. (2022). Multiple daily brood cells define the fecundity of *Osmia lignaria* bees in a semi-natural setting. *Apidologie*, *53*(5), 54. https://doi.org/10.1007/s13592-022-00965-y

Sponsler, D. B., Hamilton, M., Wiesneth, M., & Steffan-Dewenter, I. (2024). Pollinator competition and the contingency of nectar depletion during an early spring resource pulse. *Ecology and Evolution*, *14*(6), e11531. https://doi.org/10.1002/ece3.11531

Sponsler, D., Iverson, A., & Steffan-Dewenter, I. (2023). Pollinator competition and the structure of floral resources. *Ecography*, *2023*(9), e06651. https://doi.org/10.1111/ecog.06651

Strelin, M. M., & Aizen, M. A. (2018). The interplay between ovule number, pollination and resources as determinants of seed set in a modular plant. *PeerJ*, *6*, e5384. https://doi.org/10.7717/peerj.5384

Stuligross, C., & Williams, N. M. (2020). Pesticide and resource stressors additively impair wild bee reproduction. *Proceedings of the Royal Society B: Biological Sciences*, *287*(1935), 20201390. https://doi.org/10.1098/rspb.2020.1390

Teixido, A. L., Barrio, M., & Valladares, F. (2016). Size matters: Understanding the conflict faced by large flowers in mediterranean environments. *The Botanical Review*, *82*(2), 204–228. https://doi.org/10.1007/s12229-016-9168-8

Tremayne, M. A., & Richards, A. J. (2000). Seed weight and seed number affect subsequent fitness in outcrossing and selfing *Primula* species. *New Phytologist*, *148*(1), 127–142. https://doi.org/10.1046/j.1469-8137.2000.00738.x

Vilchez-Russell, K. A., & Rafferty, N. E. (2024). Effects of heat shocks, heat waves, and sustained warming on solitary bees. *Frontiers in Bee Science*, *2*, 1392848. https://doi.org/10.3389/frbee.2024.1392848

Walsh, R. P., Arnold, P. M., & Michaels, H. J. (2014). Effects of pollination limitation and seed predation on female reproductive success of a deceptive orchid. *AoB PLANTS*, *6*, plu031. https://doi.org/10.1093/aobpla/plu031

Walters, J., Barlass, M., Fisher, R., & Isaacs, R. (2024). Extreme heat exposure of host plants indirectly reduces solitary bee fecundity and survival. *Proceedings of the Royal Society B: Biological Sciences*, *291*(2025), 20240714. https://doi.org/10.1098/rspb.2024.0714

Whittle, C. A., Otto, S. P., Johnston, M. O., & Krochko, J. E. (2009). Adaptive epigenetic memory of ancestral temperature regime in *Arabidopsis thaliana*. *Botany*, *87*(6), 650–657. https://doi.org/10.1139/B09-030

Wong, L. H., & Forrest, J. R. K. (2021). The earlier the better? Nesting timing and reproductive success in subalpine cavity-nesting bees. *Journal of Animal Ecology*, *90*(5), 1353–1366. https://doi.org/10.1111/1365-2656.13460

Yates, C. J., Elliott, C., Byrne, M., Coates, D. J., & Fairman, R. (2007). Seed production, germinability and seedling growth for a bird-pollinated shrub in fragments of kwongan in south-west Australia. *Biological Conservation*, *136*(2), 306–314. https://doi.org/10.1016/j.biocon.2006.12.003

Zhao, Z., Lu, N., & Conner, J. K. (2016). Adaptive pattern of nectar volume within inflorescences: Bumblebee foraging behavior and pollinator-mediated natural selection. *Scientific Reports*, *6*(1), 34499. https://doi.org/10.1038/srep34499

Zhou, X.-H., & He, W.-M. (2020). Climate warming facilitates seed germination in native but not invasive *Solidago canadensis* populations. *Frontiers in Ecology and Evolution*, *8*, 595214. https://doi.org/10.3389/fevo.2020.595214

Zi, H., Jing, X., Liu, A., Fan, X., Chen, S.-C., Wang, H., & He, J.-S. (2023). Simulated climate warming decreases fruit number but increases seed mass. *Global Change Biology*, *29*(3), 841–855. https://doi.org/10.1111/gcb.16498
